# Chemoattractant Signaling between Tumor Cells and Macrophages Regulates Cancer Cell Migration, Metastasis and Neovascularization

**DOI:** 10.1371/journal.pone.0006713

**Published:** 2009-08-21

**Authors:** Chad E. Green, Tiffany Liu, Valerie Montel, Gene Hsiao, Robin D. Lester, Shankar Subramaniam, Steven L. Gonias, Richard L. Klemke

**Affiliations:** 1 Department of Pathology and Moores Cancer Center, University of California San Diego, La Jolla, California, United States of America; 2 Department of Bioengineering, University of California, San Diego, La Jolla, California, United States of America; New York University School of Medicine, United States of America

## Abstract

Tumor-associated macrophages are known to influence cancer progression by modulation of immune function, angiogenesis, and cell metastasis, however, little is known about the chemokine signaling networks that regulate this process. Utilizing CT26 colon cancer cells and RAW 264.7 macrophages as a model cellular system, we demonstrate that treatment of CT26 cells with RAW 264.7 conditioned medium induces cell migration, invasion and metastasis. Inflammatory gene microarray analysis indicated CT26-stimulated RAW 264.7 macrophages upregulate SDF-1α and VEGF, and that these cytokines contribute to CT26 migration *in vitro*. RAW 264.7 macrophages also showed a robust chemotactic response towards CT26-derived chemokines. In particular, microarray analysis and functional testing revealed CSF-1 as the major chemoattractant for RAW 264.7 macrophages. Interestingly, in the chick CAM model of cancer progression, RAW 264.7 macrophages localized specifically to the tumor periphery where they were found to increase CT26 tumor growth, microvascular density, vascular disruption, and lung metastasis, suggesting these cells home to actively invading areas of the tumor, but not the hypoxic core of the tumor mass. In support of these findings, hypoxic conditions down regulated CSF-1 production in several tumor cell lines and decreased RAW 264.7 macrophage migration *in vitro*. Together our findings suggest a model where normoxic tumor cells release CSF-1 to recruit macrophages to the tumor periphery where they secrete motility and angiogenic factors that facilitate tumor cell invasion and metastasis.

## Introduction

The propensity for tumors to progress and metastasize reflects not only the oncogenic mutations in the cancer cells but also dynamic interactions involving non-malignant cells in the tumor cell microenvironment. Non-malignant cells that infiltrate a developing cancer include fibroblasts, adipocytes, endothelial cells, perivascular cells, and immune cells, all of which may contribute to cancer progression [1]. Amongst the immune cells, macrophages have been shown to play a supportive role, promoting tumor cell survival, proliferation, and metastasis [2]. Tumor-associated macrophages (TAMs) are derived from circulating peripheral blood monocytes that are attracted to the tumor vasculature where they extravasate into the interstitium and differentiate [3]. Although homing of macrophage to tumors is poorly understood, tumor cells are known to release macrophage chemoattractants including CCL2, CCL5, CCL7, CCL8, CXCL12, VEGF and CSF-1 [4]. Compared to classically activated macrophages (M1) that function as primary effector cells in the innate immune system, M2 TAMs support tumor survival by promoting local angiogenesis and tissue remodeling, while suppressing the immune response [5]. TAMs localize to the invasive areas of the tumor where they secrete a variety of cytokines and proteases involved in tumor cell invasion and metastasis [6], [7]. In this role, TAMs actively contribute to tumor progression and the transition to malignancy that often correlates with poor clinical outcome. However, deciphering the tumor-cell chemokine networks that regulate cancer progression *in vivo* remains a major challenge.

Angiogenesis, which is critical for cancer progression, is controlled by a variety of factors known to stimulate blood vessel growth and/or maturation, including VEGF, TGF-β, EGF, bFGF and TNF-α. TAMs represent a primary source of many of these angiogenic proteins in the tumor microenvironement [8], [9], [10]. In particular, VEGF is released by TAMs and is a potent stimulus for the growth of new blood vessels, increased microvascular density, vascular disruption, and leak [11]. Blood vessels that are undergoing remodeling are porous and fragile and thus more susceptible to tumor cell intravasation [12]. Therefore, at the invasive front, TAMs may promote tumor metastasis by stimulating the formation of dense microvascular networks of leaky vessels that are permissible to tumor cell intravasation, while simultaneously activating cancer cell migration and invasion by releasing a variety of chemokines, mitogens and proteases.

In addition to the invasive front, TAMs may also localize to the avascular hypoxic core of the tumor [13], [14]. VEGF is released by TAMs in the tumor core in response to hypoxia and stabilization of HIF1α and HIF2α [15], [16]. VEGF may also be involved in recruiting TAMs to the tumor core, in addition to other poorly defined factors present in the cellular debris resulting from tumor necrosis [13]. Once localized to the core, TAMs may not only clear cellular debris but also regulate neovascularization and tumor survival. Thus, there are subsets of TAMs that are differentially distributed in the tumor microenvironment that may serve specialized roles during cancer progression [2]. We hypothesize that tumor oxygenation is a major determinant of macrophage activity in cancers. For example, in the hypoxic tumor core, TAMs may be primarily angiogenic and phagocytic, whereas under normoxic conditions at the tumor periphery, TAMs may contribute to tumor metastasis by increasing tissue remodeling and vascular density. In the latter case, VEGF release by TAMs may be regulated independently of hypoxia through interactions with invasive tumor cells or stromal cells.

Understanding the role of TAMs in cancer progression *in vivo* is complicated by the in ability to decipher the multitude of factors present in the microenvironment of the tumor. Therefore, model systems that recapitulate *in vivo* tumor cell-TAM interactions *in vitro* are necessary to help unravel the complexities of tumor progression and metastasis under defined conditions. In the present study, we developed a model system to directly investigate cytokine signaling between CT26 colon cancer cells and RAW 264.7 macrophages. Using this unique model system, we demonstrate that RAW 264.7 macrophages and CT26 tumor cells are mutually attracted to one another and that macrophages induce a highly migratory and protrusive phenotype in the tumor cells. Inflammatory gene array analysis and functional testing revealed that tumor cell-derived CSF-1 is the major chemoattractant for RAW 264.7 macrophages whereas macrophage derived SDF-1α and VEGF contribute to CT26 cancer cell invasion. Further, a total of 270 genes in RAW 264.7 macrophages and 85 genes in CT26 tumor cells were up- or down-regulated during incubation in conditioned media, suggesting that additional pathways beyond those tested are likely activated during bidirectional signaling. In chick CAMs inoculated with tumor cells, RAW 264.7 macrophages localize to the tumor periphery, where they facilitate vascular remodeling and potentiate tumor cell metastasis to the chick lungs. These results support a model in which paracrine signaling between tumor cells and macrophages regulates the localization of macrophages within the tumor and the propensity of the tumor cells to metastasize.

## Materials and Methods

### Cell lines, reagents and antibodies

CT26 mouse colon cancer line, RAW 264.7 mouse macrophage line and MDA-MB-468 breast cancer line were obtained from American Type Culture Collection (ATCC, Manassas, VA). CL16, a metastatic variant of MDA-MB-435, was derived as previously described [17]. CT26 cells were maintained in RPMI 1640 supplemented with 10% FBS, 1% penicillin-streptomycin (Invitrogen, Carlsbad CA) and 1% glutamine. RAW 264.7, MDA-MB-468 and CL16 cells were cultured in DMEM (Invitrogen, Carlsbad CA) supplemented with 10% FBS, 1% penicillin-streptomycin and 1% glutamax (Invitrogen, CA). RAW 264.7 expressing GFP were made by infecting cells with Lenti-Green supernatant (BioGenova, Rockville, MD). The highest 0.1% expressing cells were then sorted by FACS. CT26 cells expressing DsRed were generated by infecting cells with the lentivirus, pEF1-DsRed-pur, followed by selection in puromycin (1 µg/ml). Where indicated, cells were treated with blocking anti-CSF-1R mAb (AFS98, eBioscience, San Diego, CA), blocking anti-EGF-R (Millipore, Billerica, MA), recombinant mouse CSF-1 (R&D Systems, Minneapolis, MN), recombinant mouse EGF (R&D Systems, Minneapolis, MN), recombinant mouse SDF-1α (Millipore, Billerica, MA) or recombinant VEGF165 (Millipore, Billerica, MA).

### Quantitative cell migration and invasion assays

For time-lapse imaging of cell migration in co-culture, RAW 264.7-GFP were incubated on fibronectin (10 µg/ml, Sigma-Aldrich) coated Lab-Tek chamber slides (Nunc, Rochester, NY) for 30 min at 37°C prior to addition of CT26-DsRed (10^7^ cells/ml). Once CT26 were added, the chamber slide was immediately placed in an Inc-2000 Incubator System (20/20 Technology Inc., Wilmington, NC) then imaged at 20X (NA = 0.75) for 15 hrs at 4 frames/hr using a Nikon C1-Si inverted confocal microscope (Nikon Instruments Inc., Melville, NY) equipped with PMT detectors and lasers appropriate for GFP (488 nm) and DsRed (561 nm). Descanned images were acquired using Nikon EZ-C1 software then rendered for cell tracking and shape analysis using Imaris (Bitplane, Saint Paul, MN). Upon adhesion, the location coordinates of centroids for individual CT26-DsRed cells were recorded at each frame over the timecourse of migration. These coordinates were then used to create migration tracks from which the migration straightness, displacement and total distance were determined. Migration straightness is unitless value that relates the number of branch points or turns in a track to the total migration distance. Total migration path length was determined by summing migration step distances every frame throughout the video sequence. Net migration path displacement was quantitated as the direct distance between the start of migration and the end of migration. The shape index is the ratio of the major axis length over the minor axis length for individual cells tracked over the timecourse of the migration.

Chemotaxis assays were performed as previously described with minor modifications [18]. Briefly, modified Boyden chambers (Transwell, 6.5 mm diameter; Corning, Lowell, MA) containing polycarbonate membranes with 8 µm pores were coated on both sides with fibronectin (10 µg/ml, Sigma-Aldrich) for 2 h at 37°C, rinsed once with PBS, and then placed into the lower chamber containing 500 µl migration adhesion buffer (MAB; DMEM with 0.1% RIA-grade fraction V BSA (Sigma-Aldrich), 1% penicillin-streptomycin and 1% glutamine), complete media (DMEM supplemented with 10% FBS, 1% penicillin-streptomycin and 1% glutamine), conditioned media or migration buffer containing SDF-1α (100 ng/ml), VEGF165 (10 ng/ml), EGF (100 ng/ml) or CSF-1 (40 ng/ml), as indicated. Conditioned medias were collected from CT26 or RAW 264.7 cultures following 48 hrs of incubation at 37°C. Dilutions of conditioned medias were made in appropriate base culture media. Where indicated, anti-CSF-1R (20 µg/ml) and anti-EGF-R (20 µg/ml) were present in both top and bottom wells throughout the assay. Serum-starved RAW 264.7 or CT26 cells were removed from culture dishes with Hanks' balanced salt solution containing 5 mM EDTA and 25 mM Hepes, pH 7.2, and 0.01% trypsin, washed twice with migration buffer, and then resuspended in migration buffer at 10^6^ cells/ml. 10^5^ cells in 100 µl migration buffer were then added to the top of each migration chamber and allowed to migrate to the underside of the porous membrane for various times in triplicate. RAW 264.7 migrated for 24 hrs in all conditions and CT26 migrated for 3 hrs in all conditions. The nonmigratory cells on the upper membrane surface were removed with a cotton swab, and the migratory cells attached to the bottom surface of the membrane stained with 0.1% crystal violet in 0.1 M borate, pH 9.0, and 2% ethanol for 20 min at room temperature. The number of chemotaxing cells per membrane was counted using an inverted phase contrast microscope at 40X. For RAW 264.7 chemotaxis under hypoxic conditions, the lower chamber was supplemented with complete DMEM containing 10% FBS to create a chemotactic gradient. RAW 264.7 were then allowed to migrate for 24 hrs under normoxic (21% oxygen) or hypoxic (1% oxygen) conditions using an IsoTemp incubator (Fisher Scientific, Pittsburgh, PA). Migratory cells were fixed with 100% methanol for 5 min and stained with 0.1% crystal violet in 0.1 M borate, pH 9.0, 2% ethanol for 20 min. Membranes were cut from the Transwell and placed in 200 mL of 10% acid acetic to elute the stain then absorbance was read at 570 nm.

To examine macrophage invasion into collagen, CT26-DsRed or red fluorescent (580 nm excitation/605 nm emission) polystyrene microspheres (10 µm; Molecular Probes, Carlsbad, CA) were embedded in sterile filtered type 1 collagen gel (PureCol; Nutacon, Leimuiden, The Netherlands) containing 1X RPMI (Sigma-Aldrich), 25 mM sodium bicarbonate and adjusted to pH 7.4 using 0.1 M sodium hydroxide, as previously described [19]. The collagen solution containing CT26-DsRed (10^7^ cells/ml) or beads (10^7^ beads/ml) was allowed to gel in 10 µl aliquots on fibronectin (10 µg/ml, Sigma-Aldrich) coated Lab-Tek chamber slides (Nunc, Rochester, NY) for 30 min prior to addition of RAW 264.7-GFP (10^5^ cells/ml). Initial migration of RAW 264.7-GFP at the interface with the collagen drop was imaged at 20X (NA = 0.75) for 12 hrs at 4 frames/hr using a Nikon C1-Si inverted confocal microscope (Nikon Instruments Inc., Melville, NY) equipped with PMT detectors and lasers appropriate for GFP (488 nm) and DsRed (561 nm). Descanned images were acquired using Nikon EZ-C1 software then rendered for cell tracking using Imaris (Bitplane, Saint Paul, MN). Location coordinates of centroids for individual RAW 264.7-GFP were recorded at each frame over the timecourse of migration. These coordinates were then used to calculate the displacement and total distance migrated for cells within and beyond 100 µm of the collagen drop boundary. Slides were incubated for an additional 7 days then imaged using confocal microscopy (10X; NA = 0.45) at 1 µm increments throughout the entire Z-axis of the collagen drop.

### Chicken CAM Assay

The chick embryo metastasis assay was performed as previously described [20], [21]. Briefly, CT26-DsRed (2×10^6^ cells) alone or combined with RAW 264.7-GFP (2×10^5^ cells) cells were suspended on ice in Matrigel (BD Bioscience) then inoculated onto the chick chorioallantoic membrane (CAM) on developmental day 9. After 11 days, on developmental day 20, embryos were sacrificed and primary tumors were removed, imaged at 0.63X and 2X by stereomicroscopy, weighed and measured for diameter. The heart and lungs were isolated, and cell dissemination was quantified by counting cell clusters using confocal microscopy at 10X (NA = 0.45) with a step distance of 1 µm. Tumors were subsequently sectioned and placed directly onto a glass coverslip for imaging by confocal microscopy (10X; NA = 0.45). Tumors were imaged 0 to 0.5 cm from the periphery or edge (tumor periphery), 0.5 cm to 1 cm from the periphery (tumor wall) and 1.0 cm to 3 cm from the tumor periphery (tumor core). Quantitation of RAW 264.7-GFP distribution within the CT26-DsRed tumors was determined by averaging GFP pixel bit maps over a field of view to produce a mean fluorescence intensity for each region of the tumor using Image Pro Plus v4.5 (Media Cybernetics, Bethesda, MD). Brightness and contrast adjustments were made equally to all channels and did not modify the relative differences between GFP pixel intensity in various regions of the tumor.

### qPCR

CT26, MDA-MB-468 or CL16 tumor cells were incubated at 37°C in the appropriate complete medium for 24 hrs under hypoxic (1.0% oxygen) or normoxic (21% oxygen) conditions. Total RNA was then extracted using TRIzol (Invitrogen, Carlsbad, CA), according to the manufacturer recommendations. cDNA was synthesized from 2 µg of total RNA using the iScript cDNA synthesis kit (Bio-Rad Laboratories, Hercules, CA). qPCR was performed using a System 7300 instrument (Applied Biosystems, Foster City, CA) and a one-step program: 95°C, 10 min; 95°C, 30×, 60°C, 1 min for 40 cycles. CSF-1 and GAPDH mRNA levels were measured in triplicates and normalized against HPRT-1 mRNA.

### Microarray analysis

To examine inflammatory gene expression, conditioned buffers were collected from CT26 or RAW 264.7 cultures following 48 hrs of incubation at 37°C and applied to cultures of the opposing cell type for 24 hrs. mRNA was then isolated using the RNeasy kit (Quiagen, Valencia, CA), reverse transcribed using the iScript cDNA synthesis kit (Bio-Rad Laboratories, Hercules, CA) and analyzed in triplicate by hybridization to the Codelink Mammalian Inflammation Bioarray containing single stranded 30-mer oligonucleotide probes (GE Healthcare, Piscataway, NJ), according to the manufacturer recommendations. Raw gene expression for replicates was averaged and normalized using the CodeLink Gene Expression Analysis v5.0 Software (GE Healthcare, Piscataway, NJ). To determine statistically significant upregulation or downregulation of gene transcripts, we applied the Variance Modeled Posterior Inference with Regional Exponentials (VAMPIRE) microarray analysis web suite to raw transcript expression values, as previously described [22], [23]. Group-wise error associated with multiple comparisons was corrected using a conservative Bonferroni corrected threshold of 5% (alpha = 0.05). Hierarchical clustering of standardized array data was performed using dChip (distance: correlation, linkage: centroid) based on the gene ontology annotations for angiogensis, cell proliferation and chemotaxis [24]. For heatmap analysis, intensity scores were calculated based on the significance of gene up- or down-regulation, as determined by VAMPIRE analysis. These genes were then organized based on the gene ontology annotations for angiogensis, cell proliferation and chemotaxis using dChip (distance metric: correlation, linkage method: average).

### Statistical analysis

Data analysis was performed using GraphPad Prism version 4.0 software (GraphPad Software, San Diego, CA.). All data are reported as mean±SE. Nonparametric group data were analyzed by analysis of variance (ANOVA) and the Neuman-Keuls post-test. Gaussian-distributed mean values were analyzed by Student *t* test. Group comparisons were deemed significant for 2-tailed *P* values below .05.

## Results

### Migration and Morphological Analysis of CT26 Cancer Cells Co-Cultured with RAW 264.7 Macrophages

**Figure 1:**
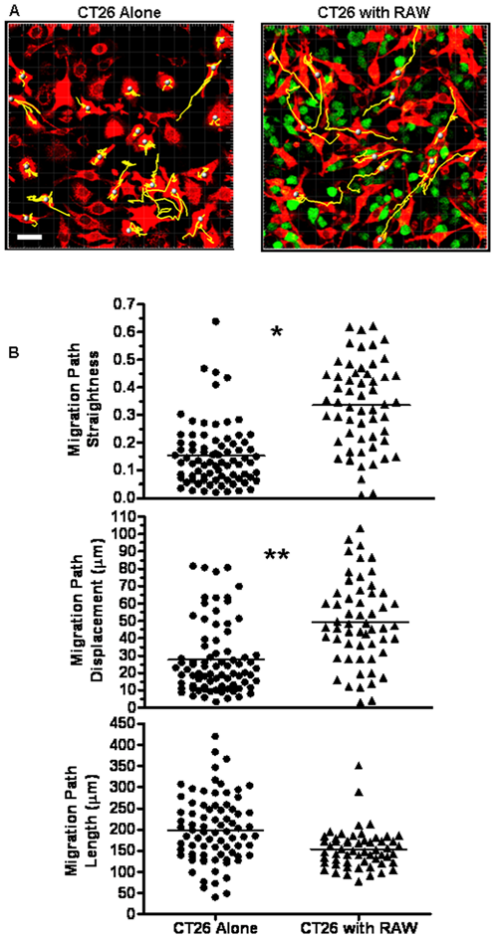
Macrophages elicit directed migration of colon cancer cells *in vitro*. A,B) CT26-DsRed (10^5^/ml) were incubated on fibronectin with RAW 264.7-GFP (10^5^/ml), as indicated, for 12 hrs at 37°C. During this time, the straightness, total length traveled and total cumulative displacement of individual CT26 cell centroids were tracked (yellow lines) at 4 frames/hr in the presence and absence of RAW 264.7 macrophages using confocal microscopy at 20X. Data is presented as individual track quantitation for 55–75 cells over 3 experiments, with average indicated by bar. Magnification scale bar represents 30 µm. * denotes significant difference in migration path straightness (p<0.0001). ** denotes significant difference in migration path displacement (p<0.0001).

We first performed a kinematic analysis of tumor cell migration behavior and determined cell shape changes in response to co-culture with macrophages. To directly examine how macrophages alter the migratory behavior and persistence of colon cancer cells, CT26 mouse colon cancer cells were imaged by confocal microscopy during co-culture for 15 hrs in the presence or absence of RAW 264.7 mouse macrophages (Fig. 1*A*). CT26 cells alone exhibited an overall migration length of 199±78 µm (average±SD) (Fig. 1*B*). This value was indistinguishable from CT26 co-cultured with RAW 264.7 cells, which exhibited a migration length of 154±46 µm. However, despite similar migration lengths, CT26 that were co-cultured with RAW 264.7 cells migrated along a significantly straighter path, with straightness values of 0.34 for CT26 co-cultured with RAW 264.7 cells compared with 0.15 for CT26 alone (Fig. 1*B*). This 2-fold increase in straightness was accompanied by a significant increase in overall displacement or persistence of migration by CT26 cells co-cultured with RAW 264.7 cells. CT26 cells co-cultured with macrophages exhibited an average displacement of 49±24 µm compared with 28±20 µm for CT26 cells alone (Fig. 1*B*). These findings suggest that the RAW 264.7 macrophages promote the migration of CT26 cells *in vitro*. Importantly, CT26 cells cultured with or without macrophages showed similar viability. In fact, analysis of tumor cell growth over several days indicated that the CT26 cells grew faster in the presence of macrophages (results not shown). Co-culturing of RAW 264.7 cells with CT26 cells did not change RAW 264.7 cell adhesion to extracellular matrix proteins. Furthermore, evaluation of RAW 264.7 cell behavior in this assay did not reveal significant differences in migration distance, displacement, straightness, or cell shape when the cells were co-cultured with CT26 cells (results not shown), suggesting the absence of chemical gradients sufficient for macrophage chemotaxis. Together, these findings indicate that RAW 264.7 macrophages induce persistent CT26 cancer cell migration on 2-D surfaces.

**Figure 2:**
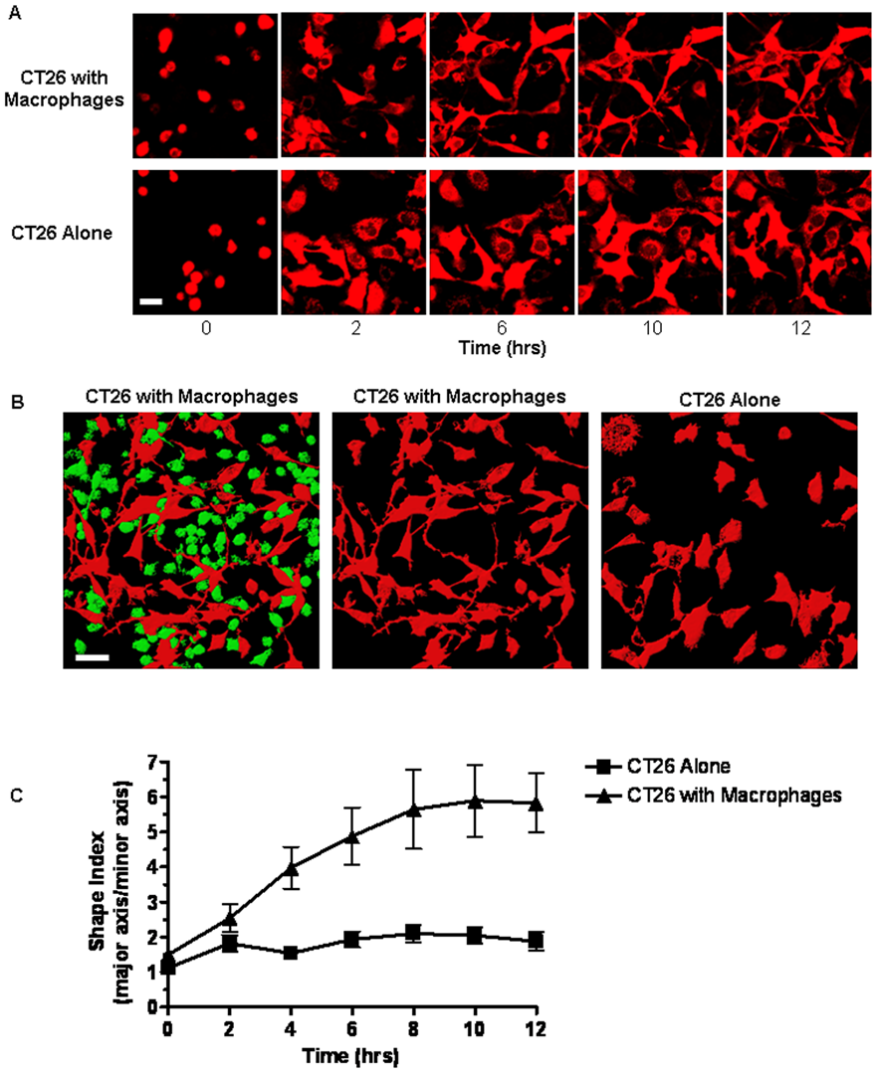
Colon cancer cells exhibit elongated protrusions when cultured with macrophages *in vitro*. DsRed-CT26 (10^5^/ml) were incubated on fibronectin with GFP-RAW 264.7 (10^5^/ml), as indicated, for 15 hrs at 37°C. Fluorescent images were acquired using confocal microscopy (20X) at 4 frames/hr. A) Time course of Ds-Red-CT26 dynamics over 12 hrs when incubated alone or with RAW 264.7-GFP macrophages. Images are representative of CT26 movement over 3 separate experiments. Magnification scale bar represents 20 µm. B) Cumulative CT26-DsRed distribution and protrusion after 15 hrs of incubation alone (right) or with RAW 264.7-GFP macrophages (left and center, with green channel turned off). Images are representative of 3 separate experiments. Magnification scale bar represents 30 µm. C) The shape index (major axis/minor axis) of CT26 cells in the presence or absence of RAW 264.7 cells was tracked over 12 hrs of migration. Data represents the average±SEM for 10 cells over 3 separate experiments.

The ability of cancer cells to form invadapodia and membrane protrusions has been linked to increased migration and tissue invasiveness [25]. Therefore, we examined CT26 cell morphology in response to co-culturing with RAW 264.7 macrophages. Within 6 hrs, co-culturing with the RAW 264.7 macrophages promoted an enhanced mesenchymal phenotype in the CT26 cells, characterized by numerous long membrane protrusions that radiated outward from the cell body (Fig. 2*A*). This morphology was sustained for greater than 12 hrs and was evident in all CT26 cells within the co-culture that were in contact with one or more macrophages (Fig. 2*B*). To determine the minimum time required for RAW 264.7 cells to elicit CT26 morphology changes, we measured the kinetics of shape change (Fig. 2*C*). Beginning with initial adhesion of cells to the dish, the major and minor axes of migrating CT26 cells were determined every 20 min throughout 12 hrs of culture with or without RAW 264.7 cells using a chamber slide and confocal microscopy. As anticipated, CT26 cells alone or in co-culture were effectively round upon initial adhesion to the dish with shape indices of ∼1. CT26 cells incubated with RAW 264.7 cells underwent rapid cell elongation, as the major axis along the polarized membrane extensions was ∼4-fold longer than the minor axis as early as 4 hrs following initial cell adhesion compared with cells cultured in the absence of RAW 264.7 macrophages (Fig. 2*C*). The shape change reached a plateau at ∼5.5 after 8 hrs of co-culture. As expected, CT26 cells that were cultured alone also exhibited an initial increase in shape extension as they adhered and spread. However, in this case, the cells only extended short protrusions and failed to elongate significantly, as the shape index only reached a maximum of ∼2 after 6 hrs of culturing (Fig. 2*C*). Taken together, quantitative analysis of CT26 cell migration and morphology dynamics indicate that RAW 264.7 macrophages elicit a rapid and sustained increase in cell migration that is associated with increased formation of membrane protrusions.

### RAW 264.7 Macrophages and CT26 Tumor Cells Release Soluble Chemotactic Factors that Promote Reciprocal Chemotaxis

**Figure 3:**
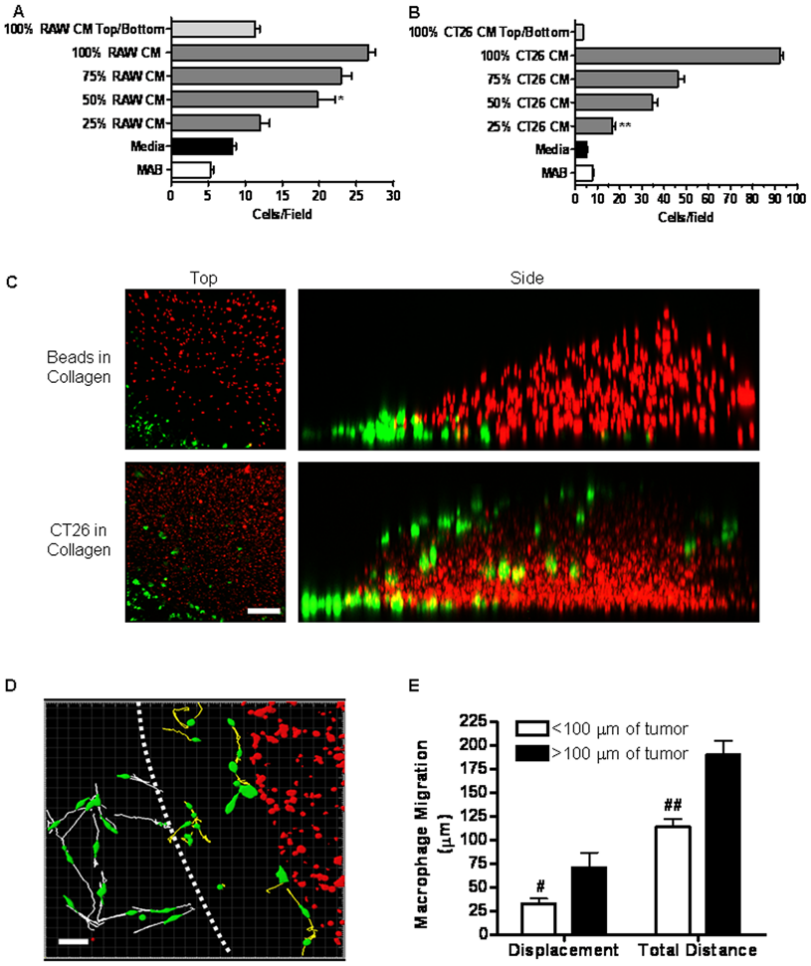
Macrophages and colon cancer cells chemotax to soluble cues. A) CT26 (10^5^/ml) were added to the upper of a Boyden chamber with RAW 264.7 conditioned media, control buffer or complete DMEM added to the lower well. CT26 were allowed to migrate for 3 hrs at 37°C prior to staining and quantitation of chemotaxis. Data represents the average±SEM for 15–30 randomly selected fields over 3–6 separate experiments. * denotes significance between 50% RAW CM and media control (p<0.001) and 50% RAW CM and 100% RAW CM top/bottom (p<0.001). B) RAW 264.7 (10^5^/ml) were added to the upper well of a Boyden chamber with CT26 conditioned media, control buffer or complete DMEM added to the lower well. RAW 264.7 were allowed to migrate for 24 hrs at 37°C prior to staining and quantitation of chemotaxis. Data represents average±SEM for 15–30 randomly selected fields over 3–6 separate experiments. ** denotes significance between 25% CT26 CM and media control (p<0.001) and 25% CT26 CM and 100% CT26 CM top/bottom (p<0.001). C) GFP-RAW 264.7 (10^5^/ml) were incubated on fibronectin coated chamber slides with 10 µl collagen drops containing Ds-Red-CT26 (10^7^/ml) or 10 µm red fluorescent beads (10^7^/ml). Macrophage invasion into the tumor embedded or bead embedded collagen drop was imaged after 7 days at 10X by confocal microscopy. Side view and top view images are representative of the average macrophage response over 6 collagen tumors. Magnification scale bar for top view images represents 200 µm. D) The interface between macrophages and the collagen tumor drop was imaged using confocal microscopy (20X) for 12 hrs at 4 frames/hr following addition of RAW 264.7 to the chamber slide. Over this time, the dynamics of macrophage migration was quantitated by tracking individual cell centroids at 4 frames/hr. Macrophages initiating migration within 100 µm of the tumor boundary (dashed white line) exhibit yellow tracks. Macrophages initiating migration beyond 100 µm of the tumor boundary exhibit white tracks. Image is representative of the average macrophage response to 6 separate collagen tumors. Magnification scale bar represents 30 µm. E) Track displacement and total track length was quantitated for macrophage migration within and beyond 100 µm of the collagen tumor boundary. Data represents the average±SEM for 15 macrophages within 100 µm and 15 macrophages beyond 100 µm of the collagen tumor boundary. #denotes a significant difference in migration path displacement (p<0.05). ##denotes a significant difference in total migration path length (p<0.0001).

We next examined whether induction of an invasive phenotype in CT26 cells by RAW 264.7 cells was due to a direct interaction with the macrophages or a response to soluble chemotactic factors released by the macrophages using a standard Boyden chamber assay [18]. CT26 cells demonstrated a dose-dependent and robust chemotactic response towards a gradient of RAW 264.7 cell conditioned media (CM), whereas exposure to control basal medium alone did not induce a migratory response (Fig. 3*A*). Importantly, cell migration was predominantly directional toward the concentration gradient, as tumor cell migration was reduced by ∼60% when RAW 264.7 cell CM was added uniformly to both top and bottom chambers (Fig. 3*A*). These findings indicate that RAW 264.7 macrophages release soluble factors that promote directional migration of CT26 colon cancer cells.

RAW 264.7 macrophages exhibited a robust and dose-dependent chemotactic response to a gradient of CT26 cell CM (Fig. 3*B*). Indeed, the extent of cell migration to undiluted CM was ∼10-fold greater than basal levels of migration observed in response to either serum-containing medium or migration buffer containing only bovine albumin. RAW 264.7 cell migration was highly directional and not due to random chemokinetic movement as adding CT26 CM to both the upper and lower chambers completely abrogated the migration response (Fig. 3*B*). Interestingly, in contrast to the co-culture system where RAW 264.7 did not exhibit an increase in migration distance or displacement, the gradient of chemotactic factors in the Boyden chamber was sufficiently steep to elicit robust chemotaxis of RAW 264.7 macrophages.

To further investigate macrophage chemotaxis towards tumor cells, we developed a novel 3D migration assay in which CT26 cells were embedded in collagen gel and place drop-wise on a coverslip with RAW 264.7 cells distributed evenly along the periphery of the gel matrix. RAW 264.7 cell migration and invasion at the gel margin was tracked and quantitated using confocal microscopy and imaging software during the initial 12 hrs and after 7 days. By this approach, RAW 264.7 macrophages actively invaded the collagen gel only when tumor cells were present (Fig. 3*C*). The invasion was directional toward the tumor cells since RAW 264.7 cells showed little ability to invade collagen gels embedded with rhodamine-labeled beads in the absence of cells (Fig. 3*C*). Quantitation of the number of macrophages per microscopic field indicated an ∼8-fold increase in macrophage invasion when tumor cells were present in the collagen compared to beads alone (data not shown). These results demonstrate that CT26 cell-derived soluble attractants are required for RAW 264.7 cell chemotaxis and that adhesive interactions with the matrix alone are not haptotactic for macrophages in this model. Interestingly, RAW 264.7 macrophages at the tumor periphery showed changes in migration behavior within 12 hrs of being added to the tumor cell/collagen gels (Fig.3*D, E*). In fact, RAW 264.7 cells within ∼100 µm (yellow tracks) of the collagen/CT26 border exhibited a ∼60% reduction in the total distance of migration suggesting a decrease in random movement. Moreover, total displacement of this subpopulation was reduced by ∼50%, further supporting a decrease in random movement. Most importantly, track analysis indicated that RAW 264.7 cells within ∼100 µm rarely migrated away from the tumor. In contrast, RAW 264.7 cells beyond ∼100 µm (white tracks) of the tumor border exhibited rapid chemokinetic movement with no persistent, directional path (Fig. 3*D, E*). These data and the Transwell migration data indicate that CT26 tumor cells secrete potent chemotactic factors that attract macrophages. These findings are interesting in light of the fact that we did not detect changes in the migration behavior of RAW 264.7 cells when co-cultured directly with CT26 cells. Under these conditions it is likely that the CT26 cell-derived factors may not provide a suitable and stable gradient to direct RAW 264.7 cell migration. Alternatively, direct contact of RAW 264.7 cells with tumor cells may prevent chemotactic movement. Collectively, the data indicates that both RAW 264.7 macrophages and CT26 tumor cells release soluble chemoattractants that promote directional cell migration.

### Changes in Gene Expression Profiles Induced by Co-Culturing RAW 264.7 Macrophages and CT26 Colon Cancer Cells

While recent evidence indicates that TAMs promote tumor growth and metastasis and suppress the normal anti-tumor immune response, the mechanisms that regulate this complex process remain poorly understood [2], [26]. The results presented here support a model in which tumor cells and macrophages rapidly adopt a chemotactic phenotype due to soluble cytokines released by the opposing cell type. To elucidate critical mediators that may be involved, we examined expression of 854 inflammatory pathway genes by CT26 cancer cells and RAW 264.7 cells after exposure to migration buffer conditioned by the opposing cell type. Briefly, RAW 264.7 macrophages or CT26 tumor cells were cultured in migration buffer (media with BSA rather than serum) for 24 hrs to generate conditioned buffer (CB). RAW 264.7 cell CB was then added to cultures of serum-starved CT26 cells. Likewise, CT26 cell CB was added to cultures of serum-starved RAW 264.7 macrophages. Following incubation for 24 hrs in CB, mRNA was isolated and analyzed using the CodeLink Mammalian Inflammation Bioarray. Significant differences in gene expression were determined using VAMPIRE (Variance-Modeled Posterior Inference of Microarray Data), a Bayesian statistical method that models the dependence of measurement variance on the amplitude of gene expression [22], [23]. Rather than the *a priori*-determined fold-change cutoff as a means of examining significance of up- or downregulated gene expression, VAMPIRE accounts for the experimental false positive error rate and the relationship between signal variance and gene expression to determine a more statistically accurate model of differential gene expression. Moreover, the fold-change cutoff method alone as a differential expression test does not account for signal variance and offers no associated level of confidence [27]. Using this approach, RAW 264.7 macrophages that were exposed to CT26 cell CB upregulated 244 genes and downregulated 26 genes based on fold-change cutoffs of 1.2 and 0.27, respectively (Supplemental Table S1). CT26 tumor cells that were exposed to RAW 264.7 cell CB upregulated 69 genes and downregulated 16 genes with fold-change cutoffs of 1.4 and 0.005, respectively (Supplemental Table S2). Hierarchical clustering and annotation of differentially expressed transcripts indicates gene signatures associated with a variety of ontology groups, including cell proliferation, chemotaxis, and angiogenesis for both CT26 and RAW 264.7 in conditioned buffers (Supplemental Figs. S1 *A–D* and S2*A–D*). A partial list of the most highly up- or down-regulated genes from these groups, as well as genes relevant to this model, is presented in Table 1 and Table 2. For example, RAW 264.7 cells stimulated with tumor cell CB up-regulated several angiogenic factors (VEGF-A, HIF1α and TGF-β) and chemoattractants (CXCL12 and CXCL2) (Table 1). It is notable that upregulation of CD14, TGF-β, CCR1, IL-18 and CXCL12 have previously been associated with the phenotypic profile of TAMs [5], [28]. Genes downregulated include uPA, lymphotoxin B, CCL4, MMP-2 and TNF-α (Table 1). Among these, TNF-α is reported to be poorly expressed in TAMs and may be pro-apoptotic to tumor cells [5]. Taken together, CT26 CB stimulates RAW 264.7 macrophages to produce proliferative, angiogenic and motility factors which may impact cancer progression.

**Table 1:** Microarray analysis of inflammatory gene expression during culture of RAW 264.7 macrophages in tumor cell conditioned buffer.

Gene	Gene Description	CB/Ctrl
uPA	Urokinase Plasminogen Activator	0.3
LTB	Lymphotoxin B	0.6
CCL4	Chemokine (C-C motif) Ligand 4	0.6
IL8Ra	Interleukin 8 Receptor, Alpha	0.6
MMP2	Matrix Metallopeptidase 2	0.6
TNF-a	Tumor Necrosis Factor alpha	0.8
CCR1	Chemokine (C-C motif) Receptor 1	1.2
IL18	Interleukin 18	1.2
Vim	Vimentin	1.2
RelA	V-rel Reticuloendotheliosis Viral Oncogene Homolog A	1.2
CtsD	Cathepsin D	1.3
TGFb1	Transforming Growth Factor, Beta 1	1.3
Casp8	Caspase 8	1.3
Arrb2	Beta 2 Arrestin	1.3
Vav1	Vav 1 Oncogene	1.3
IL16	Interleukin 16	1.4
ILK	Integrin Linked Kinase	1.4
XCR1	Chemokine (C motif) Receptor 1	1.4
IL11Ra1	Interleukin 11 Receptor, Alpha Chain 1	1.4
ITGb2	Beta 2 Integrin	1.5
CXCL2	Chemokine (C-X-C motif) Ligand 2	1.6
CSF2Rb2	Colony Stimulating Factor 2 Receptor, Beta 2	1.6
IL1R-L1	Interleukin 1 Receptor-like 1	1.7
IL15Ra	Interleukin 15 Receptor, Alpha Chain	1.7
NCAM1	Neural Cell Adhesion Molecule-1	1.8
VEGF-A	Vascular Endothelial Growth Factor A	1.8
CD14	CD14 Antigen	1.9
CXCL12	Chemokine (C-X-C motif) Ligand 12	2.3

CT26 tumor cell conditioned buffer was applied to RAW 264.7 cells for 24 hrs prior to mRNA isolation and analysis using the Codelink Mammalian Inflammation Bioarray. Significance of transcript upregulation or downregulation was determined using the VAMPIRE statistical algorithm. Data represents the fold change in transcript expression from 3 separate experiments. Of the 270 genes detected in RAW 264.7 cells (Supplemental Table S1), the most highly upregulated or downregulated genes associated with migration, proliferation and angiogenesis are presented.

**Table 2:** Microarray analysis of inflammatory gene expression during culture of CT26 tumor cells in macrophage conditioned buffer.

Gene	Gene Description	CB/Ctrl
FGF10	Fibroblast Growth Factor 10	0.3
CD80	CD80 Antigen	0.6
HSP1A	Heat Shock Protein 1A	0.6
SOCS1	Suppressor of Cytokine Signaling 1	0.7
CD14	CD14 Antigen	0.7
VEGF-A	Vascular Endothelial Growth Factor A	1.5
RelA	V-rel Reticuloendotheliosis Viral Oncogene Homolog A	1.5
TGFb3	Transforming Growth Factor, Beta 3	1.6
CXCL10	Chemokine (C-X-C motif) Ligand 10	1.6
TGFb1	Transforming Growth Factor, Beta 1	1.8
ARHGEF2	Rho/Rac Guanine Nucleotide Exchange Factor 2	1.8
RalGDS	Ral Guanine Nucleotide Dissociation Stimulator	1.9
NFkBIb	NFkB Inhibitor Beta	1.9
Arrb2	Beta 2 Arrestin	2.0
NFkB2	NFkB 2	2.1
Il15Ra	Interleukin 15 Receptor, Alpha Chain	2.2
RelB	Avian Reticuloendotheliosis Viral Oncogene Related B	2.4
CSF1	Colony Stimulating Factor 1	2.6
NFkBIa	NFkB Inhibitor Alpha	2.7
VCAM1	Vascular Cell Adhesion Molecule 1	3.0
CXCL1	Chemokine (C-X-C motif) Ligand 1	3.1
CCL2	Chemokine (C-C motif) Ligand 2	3.2
MMP10	Matrix Metallopeptidase 10	3.3
CCL20	Chemokine (C-C motif) Ligand 20	3.8
NFkBIe	NFkB Inhibitor Epsilon	4.8
CSF2	Colony Stimulating Factor 2	5.1
CXCL2	Chemokine (C-X-C motif) Ligand 2	6.7
INFa9	Interferon Alpha 9	9.3

RAW 264.7 conditioned buffer was applied to CT26 cells for 24 hrs prior to mRNA isolation and analysis using the Codelink Mammalian Inflammation Bioarray. Significance of transcript upregulation or downregulation was determined using the VAMPIRE statistical algorithm. Data represents the fold change in transcript expression from 3 separate experiments. Of the 85 genes detected in CT26 cells (Supplemental Table S2), the most highly upregulated or downregulated genes associated with migration, proliferation and angiogenesis are presented.

CT26 cells also exhibited relevant changes in chemokine profiling when incubated with RAW 264.7 CB (Table 2). Impacted genes include VCAM-1, VEGF-A, TGF-β and CXCL1, all of which are reported to be indicative of a metastatic phenotype [29], [30], [31], [32]. In addition, GM-CSF, matrix metalloproteinase (MMP)-10, and the chemokines CXCL10, CCL2, CCL20 and CXCL2 were also up-regulated (Table 2). Most notably, CT26 cells upregulated CSF-1 ∼2.6-fold in response to RAW 264.7 cell CB. CSF-1 has been shown to be a major chemoattractant for macrophages, linked to TAM regulation of cancer progression in animals [33], [34]. Taken together, the chemokine expression profile for both RAW 264.7 and CT26 cells reflect transcriptional signatures predictive of immune modulation, angiogenesis and increased tumor cell and macrophage migration.

### SDF-1α, VEGF and CSF-1 are Key Regulators of Tumor Cell and Macrophage Chemotaxis

**Figure 4:**
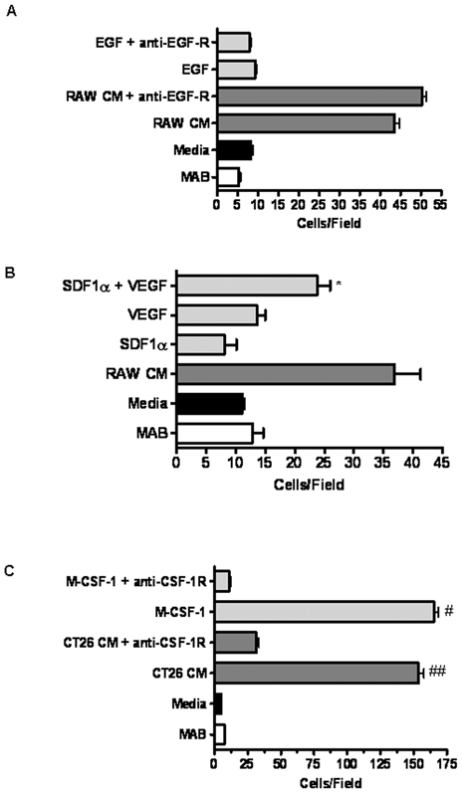
CSF-1, VEGF and SDF-1α mediate reciprocal chemotaxis between macrophages and tumor cells. A) CT26 (10^5^/ml) were added to the upper of a Boyden chamber with RAW 264.7 conditioned media, 100 ng/ml EGF in migration buffer, control migration buffer or complete DMEM added to the lower well. Anti-EGFR was present in both the top and bottom wells, as indicated, throughout the experiment. CT26 were allowed to migrate for 3 hrs at 37°C prior to staining and quantitation of chemotaxis. Data represents the average±SEM for 15–30 randomly selected fields over 3–6 separate experiments. B) CT26 (10^5^/ml) were added to the upper of a Boyden chamber with 100 ng/ml SDF-1α, 10 ng/ml VEGF, SDF-1α+VEGF, RAW 264.7 conditioned media, control migration buffer or complete DMEM added to the lower well. SDF-1α and VEGF were suspended in serum-free migration buffer. CT26 were allowed to migrate for 3 hrs at 37°C prior to staining and quantitation of chemotaxis. Data represents the average±SEM for 15–30 randomly selected fields over 3–6 separate experiments. * denotes significance between SDF-1α with VEGF compared to SDF-1α (p<0.05) or VEGF alone (p<0.05). C) RAW 264.7 (10^5^/ml) were added to the upper well of a Boyden chamber with 40 ng/ml CSF-1, CT26 conditioned media, control buffer or complete DMEM added to the lower well. CSF-1 was suspended in serum-free migration buffer. Anti-CSF-1R was present in both top and bottom wells, as indicated, throughout the assay. RAW 264.7 were allowed to migrate for 24 hrs at 37°C prior to staining and quantitation of chemotaxis. Data represents average±SEM for 15–30 randomly selected fields over 3–6 separate experiments. #denotes significance between CSF-1 and CSF-1+anti-CSF-1R (p<0.0001). ##denotes significance between CT26 CM and CT26 CM+anti-CSF-1R (p<0.0001).

We next sought to determine the primary macrophage-derived chemokines responsible for inducing tumor cell migration. Although many of the chemokines released by RAW 264.7 cells may contribute to tumor cell migration, we chose to investigate SDF-1α and VEGF since previous reports have linked these factors to cancer malignancy and both factors were significantly up-regulated in RAW 264.7 cells exposed to CT26 CB [35], [36]. Though absent in the microarray, we also investigated the role of EGF in this response because release of this growth factor by TAMs has previously been linked to cancer cell migration *in vitro* and *in vivo* [34], [37]. However, EGF failed to induce CT26 migration (Fig. 4*A*). Moreover, function blocking anti-EGF-R antibodies failed to block CT26 cell migration in response to RAW 264.7 cell CB (Fig. 4*A*). Thus, CT26 cell migration in response to RAW 264.7 CB is due to soluble factors other than EGF. Similarly, neither SDF-1α nor VEGF induced CT26 cell migration above basal levels when added separately to the migration chamber. However, when cells were stimulated with SDF-1α and VEGF simultaneously, tumor cell migration was increased ∼2-fold compared to SDF-1α or VEGF alone (Fig. 4*B*). Despite this increase, chemotaxis to SDF-1α and VEGF in combination is ∼35% less than chemotaxis to RAW 264.7 conditioned media, suggesting that other unidentified RAW 264.7-derived factors likely contribute to CT26 chemoattraction. Indeed, many known chemoattractants that may also be involved in CT26 migration are among the 270 inflammatory genes that were identified as being up- or down-regulated in RAW 264.7. Nonetheless, these findings indicate that SDF-1α and VEGF work synergistically to potentiate the chemotaxis of CT26 colon cancer cells.

Previous reports implicate CSF-1 secreted by breast cancer cells as the exclusive chemoattractant for TAMs [33], [34], [37]. Gene profiling of CT26 cancer cells also indicates upregulation of CSF-1 in the presence of RAW 264.7 cell CB. Therefore, we tested whether CSF-1 is responsible for the increase in RAW 264.7 cell migration in the presence of CT26 cell CB. Purified CSF-1 was potent in eliciting RAW 264.7 cell chemotaxis (Fig. 4*C*). This response was inhibited with function blocking antibodies to the CSF-1 receptor (c-fms/CSF-1R). Likewise, anti-CSF-1R antibody reduced RAW 264.7 cell migration to CT26 cell CB by ∼85%. It is important to note that the CSF-1 present in the CT26 cell CB represents basal secretion, as the conditioned media was generated by CT26 cells that had not been exposed to RAW 264.7 cells *a priori*. Once CT26 cells are exposed to RAW 264.7 CB, transcript analysis indicates that CT26 upregulate CSF-1 ∼2.6-fold above basal secretion levels (Table 1). Taken together, the data indicates that chemotaxis of RAW 264.7 macrophages to quiescent CT26 cells is robust and occurs primarily in response to tumor cell-secreted CSF-1, which can be further enhanced by exposure to macrophage-derived products.

### RAW 264.7 Macrophages Promote CT26 Tumor Formation, Metastasis, Vascular Density, and Vascular Disruption In Vivo

**Figure 5:**
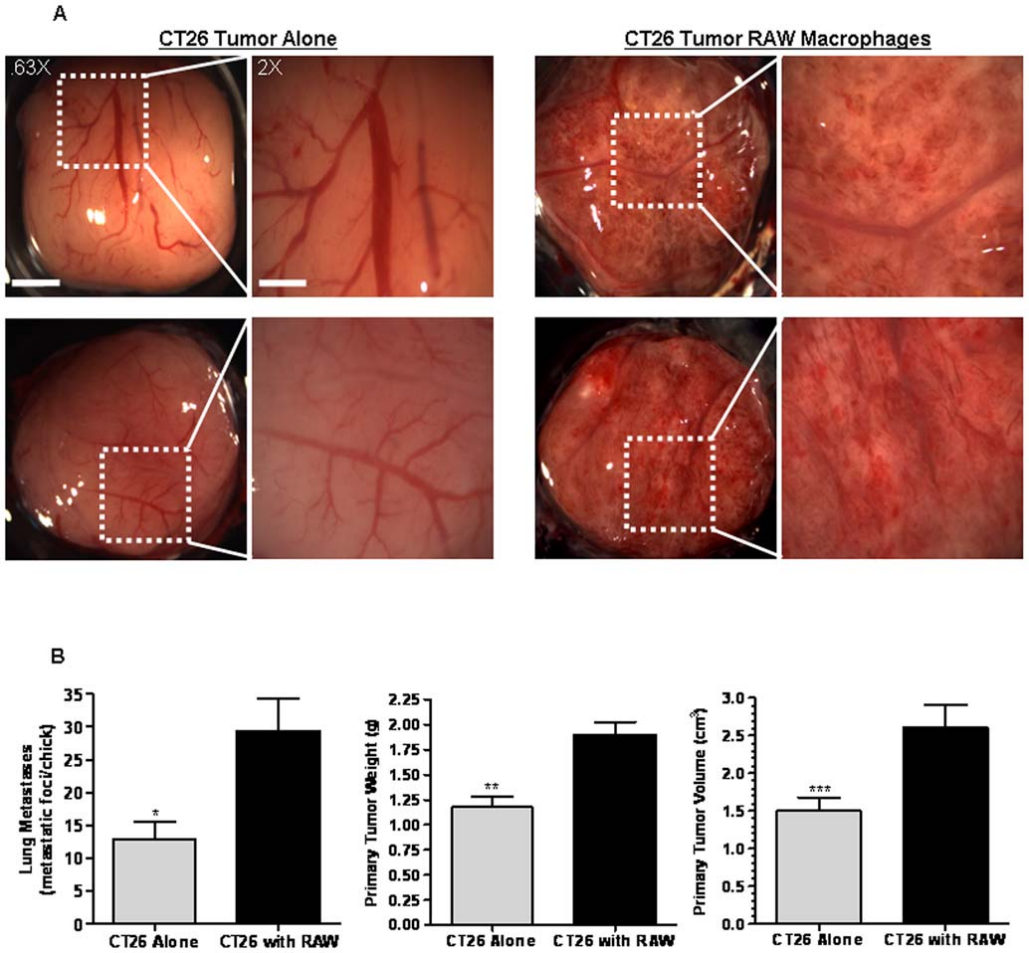
RAW 264.7 macrophages promote CT26 tumor formation, metastasis and neovascularization in the chick CAM. A) CT26-DsRed (1.8×10^6^ cells) alone or together with RAW 264.7-GFP (2×10^5^ cells) were suspended in Matrigel then inoculated onto the chick CAM. After 11 days, the primary tumors were imaged at 0.63X and 2X by stereomicroscopy then removed, weighed and measured. Images are representative of tumors from 7–10 separate experiments. Magnification scale bar for the 0.63X images represents 3 mm. Magnification scale bar for the 2X images represents 1 mm. B) CT26 metastasis was quantitated by counting the cell clusters present in the chick lungs using confocal microscopy at 10X. Data represents the average±SEM for 14–16 lungs over 4–6 separate experiments. * denotes significance between primary tumors with CT26 alone compared to primary tumors with CT26 and RAW 264.7 (p<0.01). Explanted tumors were also analyzed for weight and volume. Data represents the average±SEM for 7–10 tumors over 4–6 separate experiments. ** denotes significance in weight between primary tumors with CT26 alone compared to primary tumors with CT26 and RAW 264.7 (p<0.001). *** denotes significance in volume between primary tumors with CT26 alone compared to primary tumors with CT26 and RAW 264.7 (p<0.001).

Our accumulative data indicate that the chemokine and growth factor networks established between RAW 264.7 macrophages and CT26 tumor cells results in the induction of cell migration and the release of angiogenic factors. Next, we tested whether RAW 264.7 macrophages potentiate CT26 cancer progression *in vivo*. For these studies, we utilized the common chicken egg chorioallantoic membrane (CAM) assay which allows for the evaluation of tumor formation, angiogenesis, and cell metastasis to the lungs in 11 days (Lester etal JCB 2007; Kim et al Cell 1998). CT26 tumor cells, in the presence or absence of RAW 264.7 macrophages, were suspended in Matrigel and inoculated onto the CAM surface. After 11 days *in vivo*, the tumors were imaged then excised from the CAM to assess tumor size and vascularization. The chick lungs were also removed and the extent of tumor cell dissemination was determined by counting the number of tumor nodules. Strikingly, CT26 tumors that developed in the presence of RAW 264.7 macrophages exhibited a significant increase in tumor size, vascular density, vascular disruption and leak compared to control CT26 tumors without RAW 264.7 cells (Fig. 5*A and B*). Moreover, CT26 cell metastasis to the lungs was increased ∼2-fold in the presence of RAW 264.7 macrophages compared to control CT26 tumors (Fig. 5*A and B*). CT26 cells were not observed in the chick heart under any condition (results not shown). The dramatic increase in small microvessel density and the highly disorganized and disrupted nature of the vessels made quantification of typical vascular perimeters (branch points, and length) impossible (Fig. 5*A*). This vascular phenotype was observed in greater than 80% of the CT26 tumors with RAW 264.7 cells compared to less than 10% of control tumors. Associated with the vascular disruption and increased tumor mass induced by RAW 264.7 cells was a ∼2.5-fold increase in the ability of CT26 cells to metastasize to the lungs (Fig. 5*B*). Thus, RAW 264.7 macrophages induce a highly malignant phenotype in CT26 cells and contribute to their dissemination to the lungs. Taken together, our *in vitro* and *in vivo* data indicate that RAW 264.7 macrophages strongly increase the migratory and metastatic properties of CT26 colon cancer cells in a manner that is associated with increased vascular density and vascular disruption.

### Analysis of Macrophage Localization in CT26 tumors In Vivo

**Figure 6:**
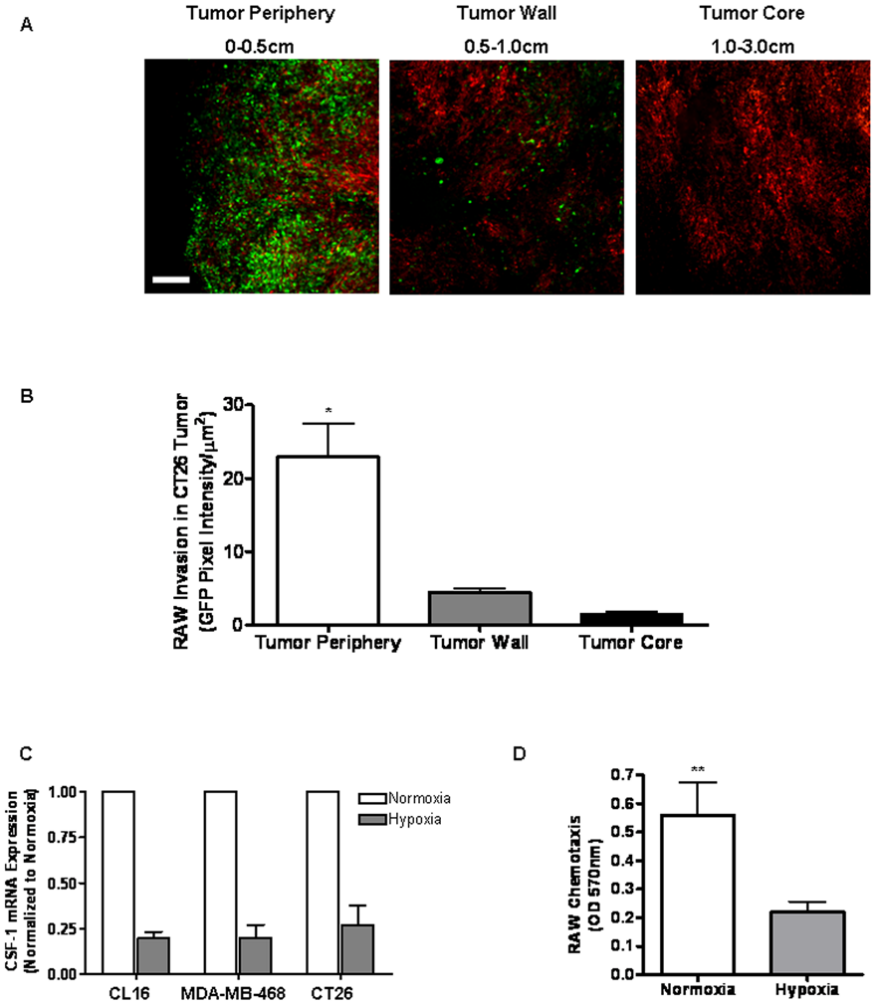
Analysis of Macrophage Localization in CT26 tumors *In Vivo*. A) CT26-DsRed (1.8×10^6^ cells) together with RAW 264.7-GFP (2×10^5^ cells) were suspended in Matrigel then inoculated onto the CAM of chick embryos for 11 days. After 11 days, the primary tumors were sectioned and imaged at 10X across the Z-axis using confocal microscopy. Tumors were imaged 0 to 0.5 cm from the periphery (Tumor Periphery), 0.5 cm to 1 cm from the periphery (Tumor Wall) and 1.0 cm to 3 cm from the tumor periphery (Tumor Core). Images are representative of tumors from 7–10 separate experiments. Magnification scale bar represents 200 µm. B) RAW 264.7-GFP distribution within the CT26-DsRed tumors was determined by averaging pixel bit maps to produce a mean fluorescence intensity for each region of the tumor. Data represents the average±SEM for RAW 264.7-GFP distribution in tumors from 3 separate experiments. * denotes significance in pixel intensity between the tumor periphery region and the tumor wall region (p<0.001). C) CT26 and human breast cancer lines CL16, metastatic variant of MDA-MD-435 and MDA-MB-468 were incubated in 1% O_2_ for 24 hrs in standard media prior to mRNA extraction and qPCR. CSF-1 mRNA expression under hypoxic conditions is presented as a percentage of basal expression under normoxic conditions. HPRT-1 was used to normalize by global gene expression. Data represents the average±SEM from 3 separate experiments. D) RAW 264.7 (2×10^5^ cells) were added to the upper well of a Boyden chamber with complete DMEM added to the lower well. RAW 264.7 were allowed to migrate for 24 hrs at 37°C under normoxic (21% oxygen) or hypoxic (1% oxygen) conditions prior to crystal violet staining and quantitation of chemotaxis by absorbance at 570 nm. Data represents average±SEM for 15–30 randomly selected fields over 3 separate experiments. ** denotes significance between RAW 264.7 chemotaxis during hypoxic and normoxic conditions (p<0.05).

Published data suggests that macrophages are recruited to sites of solid tumor formation *in vivo* in response to soluble cues [13], [33]. However, the location and distribution of macrophages within the tumor proper and surrounding microenvironment is still not clear. Existing work indicates that macrophages target the outer perimeter of the tumor where they contribute to cancer invasion and angiogenesis [7]. However, TAMs also may localize within the tumor interior where they are believed to facilitate the removal of dead cells and debris [13]. To examine the distribution of RAW 264.7 macrophages in the CT26 tumor in the CAM model, macrophages and CT26 cells were transfected with GFP and DsRed, respectively, and co-transplanted onto chick CAMs. Following 11 days of incubation, the tumors were surgically removed, weighed, measured, and then dissected in half before being placed directly onto the imaging chamber. Confocal images were then collected sequentially from the tumor border to the central core of the tumor using a 10X objective. Quantitation of macrophage distribution was determined by calculating average GFP pixel intensity present in each tumor region. As shown in Fig. 6, RAW 264.7 macrophages localized primarily within the first 0.5 cm of the tumor edge with few macrophages observed in the region 0.5–1 cm from the tumor border. Moreover, no macrophages were observed in the center of the tumor, suggesting specific migration of macrophages towards CT26 cells residing at or near the tumor border.

### Tumor Cell Hypoxia Regulates Expression of CSF-1

**Figure 7:**
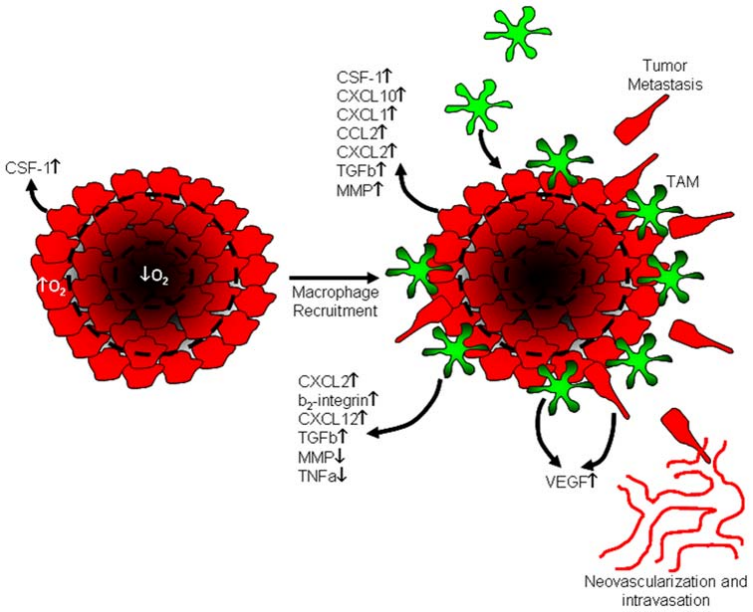
Proposed model for the role of tumor associated macrophages in cancer progression. During tumor growth, macrophages home to normoxic regions at the tumor periphery in response to secreted CSF-1. The macrophages in turn release soluble chemokines that stimulate the tumor cells to release more CSF-1 creating a localized high concentration of CSF-1 that promotes further macrophage infiltration and survival at the tumor periphery. The close proximity of macrophages and tumor cells establishes a paracrine chemokine network at the tumor margin that results is at least two major outcomes. First, tumor cell migration and tissue invasion increases as the result of SDF-1α and VEGF release by tumor associated macrophages. Second, both tumor cells and macrophages are stimulated to release VEGF and TGFβ, which facilitates vessel growth, remodeling, and increased permeability. The increase in tumor cell invasiveness combined with structural changes in the surrounding vasculature provides optimal conditions for tumor cell intravasation and metastasis.

While the role of hypoxia in regulation of CSF-1 expression has not been previously determined, we hypothesized that CSF-1 levels may be higher at the tumor margin where invading cells are exposed to an oxygen-rich environment compared to the hypoxic tumor core. To investigate this hypothesis, CSF-1 mRNA levels were quantified in CT26 cells cultured for 24 hours under normoxic or hypoxic conditions. Hypoxia caused a significant reduction in CSF-1 mRNA levels compared to normoxic cells (Fig. 6*C*). Similarly, MDA-MB-468 breast carcinoma cell and metastatic CL16 cancer cells, which are derived from MDA-MB-435 cells, also demonstrated decreased CSF-1 gene expression when switched from normoxic to hypoxic conditions, suggesting that this response may be widespread amongst various types of cancer. As anticipated, hypoxic conditions did not change the level of GAPDH or HPRT-1 in the tumor cells, which served as internal controls (results not shown). Numerous chemokine and survival promoting genes have been reported to be upregulated by tumor cells exposed to hypoxic conditions [38]. Thus, we hypothesize that the selective localization of RAW 264.7 macrophages to the tumor periphery occurs in response to a CSF-1 gradient generated between hypoxic and the normoxic regions of the tumor. In support of this contention, RAW 264.7 macrophages do not migrate effectively under hypoxic conditions characteristic of the tumor core microenvironment (Fig. 6*D*). Together, the data presented here suggest a preliminary model where macrophages home to the tumor periphery during the early stages of tumor growth in response to CSF-1 released by normoxic tumor cells. This generalized model for how macrophages regulate tumor progression is the basis for ongoing research and is summarized schematically in Figure 7.

## Discussion

The present study introduces a simple and genetically tractable system to decipher the complex bidirectional chemokine signaling between macrophages and tumor cells *in vitro* and *in vivo*. Through the use of the CT26 tumor cell/RAW 264.7 macrophage combination, our objectives were to investigate the chemotactic and angiogenic nature of the interaction then perform an analysis of the inflammatory genes that are up- or down-regulated during these responses. By this approach, the expectation was that the microarray analysis would then serve as a resource for identifying and testing specific signaling pathways activated in alternative macrophage/tumor cell interactions during cancer progression. In this work we show that, (1) soluble RAW 264.7 macrophage-derived chemokines and growth factors elicit increased CT26 tumor cell chemotaxis *in vitro* and metastasis *in vivo*, (2) in response to CT26 tumor cell conditioned media, RAW 264.7 macrophages adopt an alternative phenotype characterized by upregulation of numerous chemotactic cytokines and angiogenic growth factors, including TGF-β, VEGF, CXCL2 and SDF-1α, (3) in response to RAW 264.7 macrophage conditioned media, tumor cells also upregulate numerous chemotactic cytokines and angiogenic factors, including CCL2, CSF-1, CSF-2 and VEGF, (4) RAW 264.7 macrophages are highly chemotactic to tumor derived CSF-1, whereas CT26 tumor cells in this system chemotax to a combination of VEGF and SDF-1α but not EGF, (5) using the chick CAM as a model of the *in vivo* environment, RAW 264.7 macrophages localize to the tumor periphery where they potentiate CT26 tumor cell metastasis and neovascularization, (6) CT26 tumor cells exposed to hypoxia downregulate CSF-1 expression, suggesting a preliminary mechanism for macrophage homing to the tumor periphery rather than the tumor core.

The ability to use the RAW 264.7 cell line is advantageous, as these cells provide a high level of reproducibility compared to freshly isolated monocytes/macrophages and they can be grown in large numbers, which is amenable to large-scale biochemical and proteomic studies. Also, the Alliance for Cellular Signaling (AfCS; www.afcs.org) is generating a comprehensive and systematic profile of chemokine signaling using the RAW 264.7 cell line. Thus, the system wide efforts by the AfCS combined with the cancer model developed here could provide new insights into the complex communication networks that exist between macrophages and tumor cells. Moreover, the ability to extend these findings *in vivo* using the CAM cancer model and genetically tractable cell lines could help identify important signaling programs that operate in live animals. The CAM assay is widely used to study cancer progression model and others have engrafted tumor cells and exogenous immune cells onto the CAM tissue to investigate the role of MMPs in cancer and angiogenesis [39].

The current work demonstrates quantitatively that RAW 264.7 macrophages can increase CT26 cancer cell invasiveness *in vitro* and *in vivo*. This corroborates an accumulating body of evidence that macrophages contribute to cancer cell dissemination [2]. Although the precise mechanism is not understood, our findings indicate that SDF-1α and VEGF are two macrophage-derived chemokines that can increase tumor cell migration. Interestingly, activation of the migration machinery required both chemokines to be present, as neither SDF-1α nor VEGF alone could induce CT26 movement. This suggests that co-signaling from CXCR4 (SDF-1α receptor) and the tyrosine kinase VEGF receptor(s) are necessary to drive cell migration in this system. While previous work has demonstrated an important role for SDF-1α in mediating colon cancer cell migration and metastasis and VEGF has been previous linked to cell migration, to our knowledge this is the first observation that these two chemokines may be cooperative in the induction of cancer cell migration [35], [40].

Our findings indicate that CT26 colon cancer cells secrete CSF-1 which serves as strong RAW 264.7 macrophage chemoattractant. This work is in agreement with previous studies that have shown that CSF-1 secretion by breast cancer cells is a potent chemoattractant for macrophages *in vitro* and *in vivo* [33], [34], [37]. While these studies clearly show that tumor derived CSF-1 can direct macrophage migration and infiltration into the tumor, several important questions remain. What regulates CSF-1 release by tumor cells? Our findings indicate that there is a basal level of CSF-1 production by CT26 tumor cells that is sufficient to attract RAW 264.7 macrophages. This appears to be regulated by oxygen tension, as several tumor cell lines cultured under hypoxic conditions downregulated CSF-1 gene expression. To our knowledge this is the first demonstration showing that hypoxia regulates CSF-1 expression in tumor cells. Importantly, our gene array findings indicate that RAW 264.7 macrophage-derived chemokines upregulate CSF-1 production by CT26 tumor cells. This suggests that a positive feedback loop may exists between resident TAMs and tumor cells to maintain a locally high concentration of CSF-1. Such a bidirectional signal could then serve to maintain a large population of highly activated macrophages in close proximity to growing tumor cells. Therapeutic targeting of this communication network could be exploited as a means to slow cancer progression.

Where in the tumor proper do macrophages localize? Our findings and work by others indicate that RAW 264.7 macrophages can accumulate in the invasive zone of the advancing tumor [7]. This area of the tumor is thought to be well oxygenated compared to the tumor core. Combined with our result showing downregulation of CSF-1 expression by hypoxic tumor cells, we hypothesize that high oxygen tension at the tumor periphery and low oxygen tension at the tumor core create a chemoattractant gradient of CSF-1. While preliminary, this mechanism could direct macrophages to the tumor edge where they further amplify CSF-1 concentrations through positive feedback mechanisms as discussed above. In addition, RAW 264.7 macrophages show reduced ability to migrate under hypoxic conditions, which further supports macrophage localization to the tumor edge and not the hypoxic tumor core. This hypothesis is the focus of ongoing studies.

What are the consequences of macrophages accumulating at the edge of the advancing tumor? Our findings indicate that vascular remodeling and increased metastasis are important consequences of this process. We observed that the RAW 264.7 macrophages induced a dramatic increase the microvascular density surrounding the CT26 tumor. In fact, the vessels were so disrupted and leaky that it precluded direct enumeration of vessel changes. Although the angiogenic mechanisms that generate this type of response have not been fully elucidated, previous studies have shown that both tumor cells and TAMs express VEGF [35], [41]. Corroborating these studies, we found that the interaction of CT26 tumor cells and RAW 264.7 macrophages increased VEGF-A gene expression by both the CT26 cells and RAW 264.7 macrophages under normoxic conditions. This suggests that the paracrine signaling between tumor cells and TAMs may be sufficient to induce the release of angiogenic factors in the absence of hypoxia. The resulting high concentration of VEGF would be expected to drive vascular remodeling and permeability which in turn provides an environment rich for tumor growth and metastasis. In fact, disrupted neovasculature has been shown to provide portholes for invasive cancer cells to access the vascular compartment [12]. This combined with the known ability of TAMs to secrete extracellular matrix proteases leading to increased tumor cell invasion could account for the increased cell metastasis associated with TAM infiltration [42].

Based on our findings and the work of others we propose a hypothetical model in which normoxic invasive tumor cells release CSF-1 into the extracellular environment creating a chemokine gradient that immobilizes monocytes/macrophages to the invasive tumor edge (Fig. 7). This in turn stimulates the release of macrophage derived chemokines, including SDF-1α and VEGF, which increase tumor cell migration/invasion, angiogenesis, and vascular disruption. Ultimately, the destabilized vasculature combined with increased tumor cell invasion provides an environment rich for cancer metastasis. The development of relevant and tractable model systems, as described in the present study, not only improve our understanding of metastasis, but may also aid in the design and testing of therapeutics that target metastatic cells and/or the macrophages that contribute to cancer cell dissemination *in vivo*. We propose that data generated in the current study be used as a community resource for the further examination of inflammatory pathways in the other macrophage/tumor cell systems. Validation of these chemical networks will be necessary to determine if redundant mechanisms exist and therapeutics can be developed to target the spectrum of human cancers.

## Supporting Information

Figure S1 Hierarchical clustering of gene transcripts upregulated and downregulated by RAW 264.7 macrophages in CT26 conditioned buffer. CT26 conditioned buffer was applied to RAW 264.7 macrophages for 24 hrs prior to mRNA isolation and analysis using the Codelink Mammalian Inflammation Bioarray. Significance of transcript upregulation or downregulation was determined using the VAMPIRE statistical algorithm. Of the 854 inflammatory genes examined, 270 were differentially expressed (Supplementary Table S1). Hierarchical clustering of this subpopulation was performed using dChip (distance: correlation, linkage: centroid) based on the gene ontology annotations for A) cell proliferation, B) angiogenesis and C) chemotaxis. Red bars and green bars denote upregulated and downregulated genes, respectively. D) For heatmap analysis, intensity scores were calculated based on the significance of gene up- or down-regulation, as determined by VAMPIRE analysis. These genes were then organized based on the gene ontology annotations for angiogensis, cell proliferation and chemotaxis using dChip (distance metric: correlation, linkage method: average). Each column in the heatmap represents an independent replicate of RAW 264.7 in CT26 conditioned buffer or RAW 264.7 in standard control buffer, as indicated.
(2.31 MB TIF)

Figure S2 Hierarchical clustering of gene transcripts upregulated and downregulated by CT26 tumor cells in RAW 264.7 conditioned buffer. RAW 264.7 conditioned buffer was applied to CT26 tumor cells for 24 hrs prior to mRNA isolation and analysis using the Codelink Mammalian Inflammation Bioarray. Significance of transcript upregulation or downregulation was determined using the VAMPIRE statistical algorithm. Of the 854 inflammatory genes examined, 85 were differentially expressed (Supplementary Table S2). Hierarchical clustering of this subpopulation was performed using dChip (distance: correlation, linkage: centroid) based on the gene ontology annotations for A) angiogensis, B) cell proliferation and C) chemotaxis. Red bars and green bars denote upregulated and downregulated genes, respectively. D) For heatmap analysis, intensity scores were calculated based on the significance of gene up- or down-regulation, as determined by VAMPIRE analysis. These genes were then organized based on the gene ontology annotations for angiogensis, cell proliferation and chemotaxis using dChip (distance metric: correlation, linkage method: average). Each column in the heatmap represents an independent replicate of CT26 tumor cells in RAW 264.7 conditioned buffer or CT26 in standard control buffer, as indicated.
(1.95 MB TIF)

Table S1 Microarray analysis of inflammatory gene expression in RAW 264.7 macrophages during incubation in CT26 tumor cell conditioned buffer. To examine inflammatory gene expression, conditioned buffer was collected from CT26 cultures following 48 hrs of incubation at 37°C and applied to RAW 264.7 cultures for an additional 24 hrs at 37°C. mRNA was then isolated, reverse transcribed and analyzed by hybridization to the Codelink Mammalian Inflammation Bioarray (GE Healthcare, Piscataway, NJ), according to the manufacturer recommendations. Significance of transcript upregulation or downregulation was determined using the VAMPIRE statistical algorithm. Data represents the fold change in transcript expression from 3 separate experiments. Of the 854 genes assayed, 270 genes were determined to be significantly upregulated or downregulated in RAW 264.7 macrophages during culture in CT26 conditioned buffer.
(0.35 MB DOC)

Table S2 Microarray analysis of inflammatory gene expression in CT26 tumor cells during incubation in RAW 264.7 macrophage conditioned buffer. To examine inflammatory gene expression, conditioned buffer was collected from RAW 264.7 cultures following 48 hrs of incubation at 37°C and applied to CT26 cultures for an additional 24 hrs at 37°C. mRNA was then isolated, reverse transcribed and analyzed by hybridization to the Codelink Mammalian Inflammation Bioarray (GE Healthcare, Piscataway, NJ), according to the manufacturer recommendations. Significance of transcript upregulation or downregulation was determined using the VAMPIRE statistical algorithm. Data represents the fold change in transcript expression from 3 separate experiments. Of the 854 genes assayed, 85 genes were determined to be significantly upregulated or downregulated in CT26 cells during culture in RAW 264.7 conditioned buffer.
(0.12 MB DOC)
