# Supplementary figures and images for: Chemoattractant Signaling between Tumor Cells and Macrophages Regulates Cancer Cell Migration, Metastasis and Neovascularization
Source: PLoS One. 2009 Aug 21;4(8):e6713. doi: 10.1371/journal.pone.0006713 (PMC2725301; doi:10.1371/journal.pone.0006713)

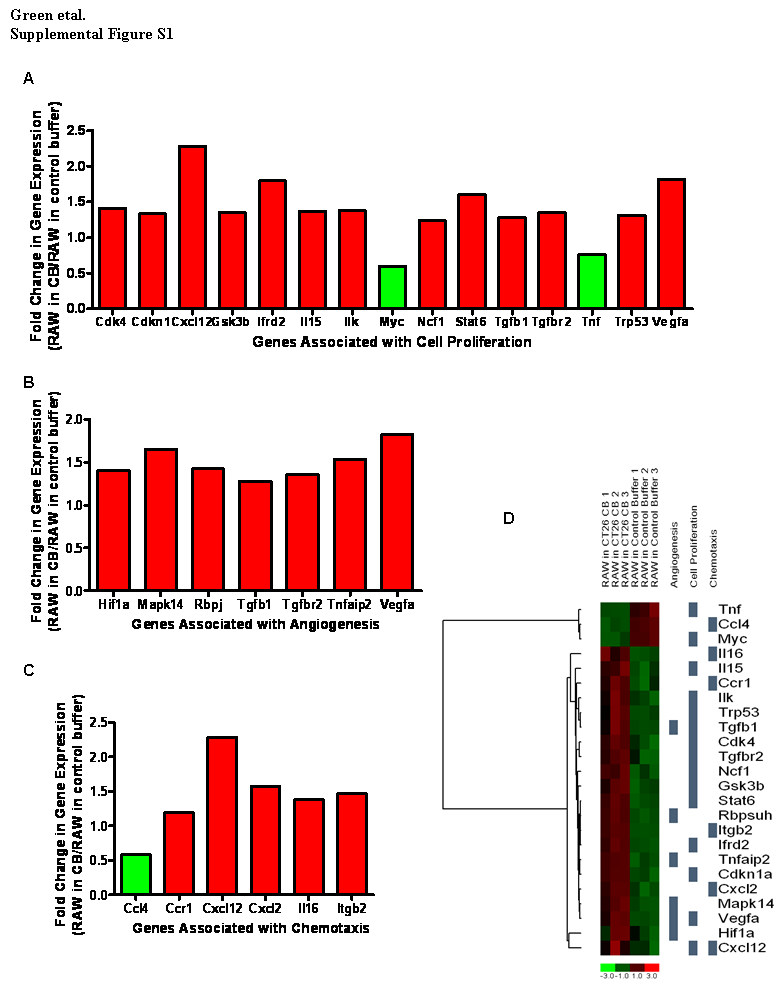

Supplement: Figure S1 — Hierarchical clustering of gene transcripts upregulated and downregulated by RAW 264.7 macrophages in CT26 conditioned buffer. CT26 conditioned buffer was applied to RAW 264.7 macrophages for 24 hrs prior to mRNA isolation and analysis using the Codelink Mammalian Inflammation Bioarray. Significance of transcript upregulation or downregulation was determined using the VAMPIRE statistical algorithm. Of the 854 inflammatory genes examined, 270 were differentially expressed (Supplementary Table S1). Hierarchical clustering of this subpopulation was performed using dChip (distance: correlation, linkage: centroid) based on the gene ontology annotations for A) cell proliferation, B) angiogenesis and C) chemotaxis. Red bars and green bars denote upregulated and downregulated genes, respectively. D) For heatmap analysis, intensity scores were calculated based on the significance of gene up- or down-regulation, as determined by VAMPIRE analysis. These genes were then organized based on the gene ontology annotations for angiogensis, cell proliferation and chemotaxis using dChip (distance metric: correlation, linkage method: average). Each column in the heatmap represents an independent replicate of RAW 264.7 in CT26 conditioned buffer or RAW 264.7 in standard control buffer, as indicated. (2.31 MB TIF) [file pone.0006713.s001.tif]

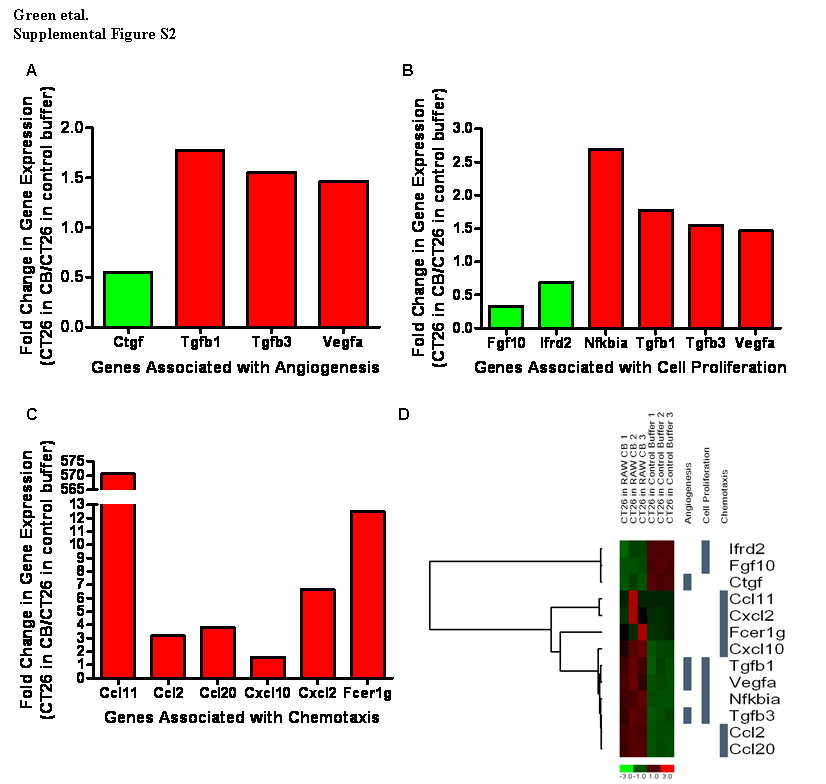

Supplement: Figure S2 — Hierarchical clustering of gene transcripts upregulated and downregulated by CT26 tumor cells in RAW 264.7 conditioned buffer. RAW 264.7 conditioned buffer was applied to CT26 tumor cells for 24 hrs prior to mRNA isolation and analysis using the Codelink Mammalian Inflammation Bioarray. Significance of transcript upregulation or downregulation was determined using the VAMPIRE statistical algorithm. Of the 854 inflammatory genes examined, 85 were differentially expressed (Supplementary Table S2). Hierarchical clustering of this subpopulation was performed using dChip (distance: correlation, linkage: centroid) based on the gene ontology annotations for A) angiogensis, B) cell proliferation and C) chemotaxis. Red bars and green bars denote upregulated and downregulated genes, respectively. D) For heatmap analysis, intensity scores were calculated based on the significance of gene up- or down-regulation, as determined by VAMPIRE analysis. These genes were then organized based on the gene ontology annotations for angiogensis, cell proliferation and chemotaxis using dChip (distance metric: correlation, linkage method: average). Each column in the heatmap represents an independent replicate of CT26 tumor cells in RAW 264.7 conditioned buffer or CT26 in standard control buffer, as indicated. (1.95 MB TIF) [file pone.0006713.s002.tif]
