# Supplementary material for: Chemoattractant Signaling between Tumor Cells and Macrophages Regulates Cancer Cell Migration, Metastasis and Neovascularization
Source: PLoS One. 2009 Aug 21;4(8):e6713. doi: 10.1371/journal.pone.0006713 (PMC2725301; doi:10.1371/journal.pone.0006713)
Supplement: Table S1 — Microarray analysis of inflammatory gene expression in RAW 264.7 macrophages during incubation in CT26 tumor cell conditioned buffer. To examine inflammatory gene expression, conditioned buffer was collected from CT26 cultures following 48 hrs of incubation at 37°C and applied to RAW 264.7 cultures for an additional 24 hrs at 37°C. mRNA was then isolated, reverse transcribed and analyzed by hybridization to the Codelink Mammalian Inflammation Bioarray (GE Healthcare, Piscataway, NJ), according to the manufacturer recommendations. Significance of transcript upregulation or downregulation was determined using the VAMPIRE statistical algorithm. Data represents the fold change in transcript expression from 3 separate experiments. Of the 854 genes assayed, 270 genes were determined to be significantly upregulated or downregulated in RAW 264.7 macrophages during culture in CT26 conditioned buffer. (0.35 MB DOC) [file pone.0006713.s003.doc]

**Green etal.**

**Supplemental Table S1**

| **Gene** | **Gene Description** | **CB/Ctrl** |
| --- | --- | --- |
| Plau | plasminogen activator, urokinase | 0.268 |
| F3 | coagulation factor III | 0.327 |
| Atf3 | activating transcription factor 3 | 0.368 |
| Gucy1b2 | guanylate cyclase 1, soluble, beta 2 | 0.483 |
| Igf1 | insulin-like growth factor 1 | 0.484 |
| Ltb | lymphotoxin B | 0.567 |
| Msx2 | homeo box, msh-like 2 | 0.57 |
| Ccl4 | chemokine (C-C motif) ligand 4 | 0.58 |
| Myc | myelocytomatosis oncogene | 0.6 |
| Il8ra | interleukin 8 receptor, alpha | 0.603 |
| Ptger4 | prostaglandin E receptor 4 (subtype EP4) | 0.629 |
| Icosl | icos ligand | 0.639 |
| Mmp2 | matrix metallopeptidase 2 | 0.642 |
| Gadd45b | growth arrest and DNA-damage-inducible 45 beta | 0.657 |
| Tnfrsf17 | tumor necrosis factor receptor superfamily, member 17 | 0.697 |
| Asb11 | ankyrin repeat and SOCS box-containing protein 11 | 0.699 |
| Jun | Jun oncogene | 0.726 |
| Ppil2 | peptidylprolyl isomerase (cyclophilin)-like 2 | 0.748 |
| Tnf | tumor necrosis factor | 0.766 |
| Slpi | secretory leukocyte peptidase inhibitor | 0.77 |
| Cd72 | CD72 antigen | 0.824 |
| Ier5 | immediate early response 5 | 0.832 |
| Fos | FBJ osteosarcoma oncogene | 0.842 |
| Ifi204 | interferon activated gene 204 | 0.846 |
| Eps8 | epidermal growth factor receptor pathway substrate 8 | 0.859 |
| Tuba4 | tubulin, alpha 4 | 1.169 |
| Mrpl3 | mitochondrial ribosomal protein L3 | 1.172 |
| Akt2 | thymoma viral proto-oncogene 2 | 1.172 |
| Cd47 | CD47 antigen (Rh-related antigen, integrin-associated signal transducer) | 1.173 |
| Hspa8 | heat shock protein 8 | 1.174 |
| Ywhae | tyrosine 3-monooxygenase/tryptophan 5-monooxygenase activation protein, epsilon polypeptide | 1.176 |
| Bfar | bifunctional apoptosis regulator | 1.176 |
| Hmga1 | high mobility group AT-hook 1 | 1.183 |
| Slc3a2 | solute carrier family 3 (activators of dibasic and neutral amino acid transport), member 2 | 1.184 |
| Sertad1 | SERTA domain containing 1 | 1.184 |
| Ccr1 | chemokine (C-C motif) receptor 1 | 1.187 |
| Bsg | basigin | 1.189 |
| Psmb9 | proteosome (prosome, macropain) subunit, beta type 9 (large multifunctional peptidase 2) | 1.19 |
| Ppil4 | peptidylprolyl isomerase (cyclophilin)-like 4 | 1.193 |
| Akt3 | thymoma viral proto-oncogene 3 | 1.193 |
| Tubb5 | tubulin, beta 5 | 1.196 |
| Cd247 | CD247 antigen | 1.198 |
| Il18 | interleukin 18 | 1.198 |
| Ccnl1 | cyclin L1 | 1.199 |
| Defb19 | defensin beta 19 | 1.2 |
| Ndufa2 | NADH dehydrogenase (ubiquinone) 1 alpha subcomplex, 2 | 1.2 |
| Pgk1 | phosphoglycerate kinase 1 | 1.201 |
| H2-T23 | histocompatibility 2, T region locus 23 | 1.202 |
| Eef1b2 | eukaryotic translation elongation factor 1 beta 2 | 1.202 |
| Bcor | Bcl6 interacting corepressor | 1.203 |
| Ywhaq | tyrosine 3-monooxygenase/tryptophan 5-monooxygenase activation protein, theta polypeptide | 1.203 |
| Traf3 | Tnf receptor-associated factor 3 | 1.204 |
| Bst1 | bone marrow stromal cell antigen 1 | 1.205 |
| Slc2a1 | solute carrier family 2 (facilitated glucose transporter), member 1 | 1.205 |
| Havcr2 | hepatitis A virus cellular receptor 2 | 1.205 |
| Jund1 | Jun proto-oncogene related gene d1 | 1.206 |
| Tnfrsf11b | tumor necrosis factor receptor superfamily, member 11b (osteoprotegerin) | 1.206 |
| Vim | vimentin | 1.208 |
| Mrpl27 | mitochondrial ribosomal protein L27 | 1.21 |
| Rela | v-rel reticuloendotheliosis viral oncogene homolog A (avian) | 1.211 |
| Hmgn2 | high mobility group nucleosomal binding domain 2 | 1.211 |
| Taf11 | TAF11 RNA polymerase II, TATA box binding protein (TBP)-associated factor | 1.211 |
| Pfkp | phosphofructokinase, platelet | 1.212 |
| Mrpl28 | mitochondrial ribosomal protein L28 | 1.212 |
| Tradd | Tradd | TNFRSF1A-associated via death domain | TNFRSF1A-associated via death domain | 1.215 |
| Bcl10 | B-cell leukemia/lymphoma 10 | 1.217 |
| Slc31a2 | solute carrier family 31, member 2 | 1.218 |
| Cyp26b1 | cytochrome P450, family 26, subfamily b, polypeptide 1 | 1.219 |
| F10 | coagulation factor X | 1.22 |
| H2afz | H2A histone family, member Z | 1.22 |
| Ppid | peptidylprolyl isomerase D (cyclophilin D) | 1.222 |
| Myd88 | myeloid differentiation primary response gene 88 | 1.222 |
| Ikbkb | inhibitor of kappaB kinase beta | 1.222 |
| Hsp90aa1 | heat shock protein 90kDa alpha (cytosolic), class A member 1 | 1.223 |
| Lamp1 | lysosomal membrane glycoprotein 1 | 1.223 |
| Nfkbil1 | nuclear factor of kappa light polypeptide gene enhancer in B-cells inhibitor-like 1 | 1.224 |
| Mrpl43 | mitochondrial ribosomal protein L43 | 1.23 |
| Mrpl4 | mitochondrial ribosomal protein L4 | 1.235 |
| Hspe1 | heat shock protein 1 (chaperonin 10) | 1.238 |
| Dad1 | defender against cell death 1 | 1.239 |
| Ncf1 | neutrophil cytosolic factor 1 | 1.239 |
| Ppia | peptidylprolyl isomerase A | 1.24 |
| Osm | oncostatin M | 1.24 |
| Mapk6 | mitogen-activated protein kinase 6 | 1.241 |
| Eef1e1 | eukaryotic translation elongation factor 1 epsilon 1 | 1.241 |
| Slc12a4 | solute carrier family 12, member 4 | 1.241 |
| Tyk2 | tyrosine kinase 2 | 1.241 |
| Ifngr2 | interferon gamma receptor 2 | 1.242 |
| Psmb8 | proteosome (prosome, macropain) subunit, beta type 8 (large multifunctional peptidase 7) | 1.244 |
| Gpx4 | glutathione peroxidase 4 | 1.246 |
| Ywhab | tyrosine 3-monooxygenase/tryptophan 5-monooxygenase activation protein, beta polypeptide | 1.247 |
| Calm2 | calmodulin 2 | 1.252 |
| Cebpd | CCAAT/enhancer binding protein (C/EBP), delta | 1.252 |
| Slc25a22 | solute carrier family 25 (mitochondrial carrier, glutamate), member 22 | 1.253 |
| Klf7 | Kruppel-like factor 7 (ubiquitous) | 1.256 |
| Eif5 | Eif5 | eukaryotic translation initiation factor 5 | eukaryotic translation initiation factor 5 | 1.26 |
| Il2rb | interleukin 2 receptor, beta chain | 1.267 |
| Capns1 | calpain, small subunit 1 | 1.268 |
| Ctsd | cathepsin D | 1.268 |
| Tlr6 | toll-like receptor 6 | 1.271 |
| Cebpb | CCAAT/enhancer binding protein (C/EBP), beta | 1.272 |
| Mcam | melanoma cell adhesion molecule | 1.277 |
| Tgfb1 | transforming growth factor, beta 1 | 1.28 |
| Eef1d | eukaryotic translation elongation factor 1 delta (guanine nucleotide exchange protein) | 1.281 |
| Mrpl36 | mitochondrial ribosomal protein L36 | 1.281 |
| Ralgds | ral guanine nucleotide dissociation stimulator | 1.283 |
| Casp8 | caspase 8 | 1.284 |
| Slc7a7 | solute carrier family 7 (cationic amino acid transporter, y+ system), member 7 | 1.284 |
| Arrb2 | arrestin, beta 2 | 1.286 |
| Bid | BH3 interacting domain death agonist | 1.287 |
| Hdgf | hepatoma-derived growth factor | 1.288 |
| Sema4a | sema domain, immunoglobulin domain (Ig), transmembrane domain (TM) and short cytoplasmic domain, (semaphorin) 4A | 1.29 |
| Igf2r | insulin-like growth factor 2 receptor | 1.295 |
| Lcp2 | lymphocyte cytosolic protein 2 | 1.296 |
| Vav1 | vav 1 oncogene | 1.296 |
| Dedd | death effector domain-containing | 1.302 |
| Map3k7ip1 | mitogen-activated protein kinase kinase kinase 7 interacting protein 1 | 1.305 |
| Il12rb1 | interleukin 12 receptor, beta 1 | 1.306 |
| Ifih1 | interferon induced with helicase C domain 1 | 1.31 |
| Il23a | interleukin 23, alpha subunit p19 | 1.311 |
| Tnfrsf1a | tumor necrosis factor receptor superfamily, member 1a | 1.312 |
| Gak | cyclin G associated kinase | 1.315 |
| Trp53 | transformation related protein 53 | 1.316 |
| Sdf4 | stromal cell derived factor 4 | 1.319 |
| Cyp27a1 | cytochrome P450, family 27, subfamily a, polypeptide 1 | 1.32 |
| Ncf2 | neutrophil cytosolic factor 2 | 1.324 |
| Tlr7 | toll-like receptor 7 | 1.325 |
| Gtf2i | general transcription factor II I | 1.325 |
| Il2rg | interleukin 2 receptor, gamma chain | 1.326 |
| Tceb3 | transcription elongation factor B (SIII), polypeptide 3 | 1.328 |
| Tcerg1 | transcription elongation regulator 1 (CA150) | 1.331 |
| Cdkn1a | cyclin-dependent kinase inhibitor 1A (P21) | 1.332 |
| 6620401K05Rik | RIKEN cDNA 6620401K05 gene | 1.337 |
| Taf10 | TAF10 RNA polymerase II, TATA box binding protein (TBP)-associated factor | 1.338 |
| Lyn | Yamaguchi sarcoma viral (v-yes-1) oncogene homolog | 1.339 |
| Ikbkg | inhibitor of kappaB kinase gamma | 1.34 |
| Psmd2 | proteasome (prosome, macropain) 26S subunit, non-ATPase, 2 | 1.341 |
| Rab11b | RAB11B, member RAS oncogene family | 1.343 |
| Tnfrsf8 | tumor necrosis factor receptor superfamily, member 8 | 1.343 |
| Eif2s3x | eukaryotic translation initiation factor 2, subunit 3, structural gene X-linked | 1.344 |
| Gsk3b | glycogen synthase kinase 3 beta | 1.351 |
| Gyk | glycerol kinase | 1.351 |
| Mrpl32 | mitochondrial ribosomal protein L32 | 1.353 |
| Hras1 | Harvey rat sarcoma virus oncogene 1 | 1.353 |
| Tgfbr2 | transforming growth factor, beta receptor II | 1.354 |
| Saa3 | serum amyloid A 3 | 1.354 |
| Akt2 | thymoma viral proto-oncogene 2 | 1.356 |
| Ly6e | lymphocyte antigen 6 complex, locus E | 1.357 |
| Stat3 | signal transducer and activator of transcription 3 | 1.358 |
| Daxx | Fas death domain-associated protein | 1.368 |
| Csnk2b | casein kinase 2, beta polypeptide | 1.369 |
| Hmox1 | heme oxygenase (decycling) 1 | 1.371 |
| Il15 | interleukin 15 | 1.372 |
| Gusb | glucuronidase, beta | 1.373 |
| Nfat5 | nuclear factor of activated T-cells 5 | 1.374 |
| Cd86 | CD86 antigen | 1.377 |
| Map2k3 | mitogen activated protein kinase kinase 3 | 1.378 |
| Taf1c | TATA box binding protein (Tbp)-associated factor, RNA polymerase I, C | 1.379 |
| Il16 | interleukin 16 | 1.379 |
| Jak3 | Janus kinase 3 | 1.38 |
| Tubb4 | tubulin, beta 4 | 1.38 |
| Ilk | integrin linked kinase | 1.381 |
| Hspa1a | heat shock protein 1A | 1.382 |
| Isgf3g | interferon dependent positive acting transcription factor 3 gamma | 1.383 |
| Dctn5 | dynactin 5 | 1.385 |
| Tgif | TG interacting factor | 1.387 |
| Nfe2l1 | nuclear factor, erythroid derived 2,-like 1 | 1.391 |
| Tfrc | transferrin receptor | 1.393 |
| Abcc1 | ATP-binding cassette, sub-family C (CFTR/MRP), member 1 | 1.393 |
| 0610006I08Rik | RIKEN cDNA 0610006I08 gene | 1.394 |
| Ticam2 | toll-like receptor adaptor molecule 2 | 1.394 |
| Itpk1 | inositol 1,3,4-triphosphate 5/6 kinase | 1.396 |
| Il7r | interleukin 7 receptor | 1.398 |
| Bcl2 | B-cell leukemia/lymphoma 2 | 1.398 |
| Hif1a | hypoxia inducible factor 1, alpha subunit | 1.398 |
| Mrpl23 | mitochondrial ribosomal protein L23 | 1.401 |
| Txnrd2 | thioredoxin reductase 2 | 1.403 |
| Traf1 | Tnf receptor-associated factor 1 | 1.405 |
| Zfp36 | zinc finger protein 36 | 1.405 |
| Nfkbib | nuclear factor of kappa light chain gene enhancer in B-cells inhibitor, beta | 1.406 |
| Tap1 | transporter 1, ATP-binding cassette, sub-family B (MDR/TAP) | 1.408 |
| Cdk4 | cyclin-dependent kinase 4 | 1.413 |
| Cd36 | CD36 antigen | 1.416 |
| Map2k1 | mitogen activated protein kinase kinase 1 | 1.421 |
| Mcfd2 | multiple coagulation factor deficiency 2 | 1.421 |
| Rbpsuh | recombining binding protein suppressor of hairless (Drosophila) | 1.421 |
| Nfkb2 | nuclear factor of kappa light polypeptide gene enhancer in B-cells 2, p49/p100 | 1.422 |
| Snrpa | small nuclear ribonucleoprotein polypeptide A | 1.424 |
| Ifnar1 | interferon (alpha and beta) receptor 1 | 1.43 |
| Cyp2s1 | cytochrome P450, family 2, subfamily s, polypeptide 1 | 1.432 |
| Stat3 | signal transducer and activator of transcription 3 | 1.434 |
| Xcr1 | chemokine (C motif) receptor 1 | 1.434 |
| Il11ra1 | interleukin 11 receptor, alpha chain 1 | 1.437 |
| Akt1 | thymoma viral proto-oncogene 1 | 1.438 |
| Tnfrsf11a | tumor necrosis factor receptor superfamily, member 11a | 1.438 |
| Map3k5 | mitogen activated protein kinase kinase kinase 5 | 1.44 |
| Dctn1 | dynactin 1 | 1.447 |
| Asns | asparagine synthetase | 1.449 |
| H2-DMa | histocompatibility 2, class II, locus DMa | 1.454 |
| Pla2g4a | phospholipase A2, group IVA (cytosolic, calcium-dependent) | 1.461 |
| Atp9b | ATPas, class II, type 9B | 1.463 |
| Bckdha | branched chain ketoacid dehydrogenase E1, alpha polypeptide | 1.469 |
| Itgb2 | integrin beta 2 | 1.469 |
| Csnk2a1 | casein kinase 2, alpha 1 polypeptide | casein kinase II, alpha 1 related sequence 4 | 1.477 |
| Nfatc3 | nuclear factor of activated T-cells, cytoplasmic, calcineurin-dependent 3 | 1.477 |
| Ldha | lactate dehydrogenase A | 1.48 |
| Traf4 | Tnf receptor associated factor 4 | 1.486 |
| Slc20a1 | solute carrier family 20, member 1 | 1.495 |
| Nme1 | expressed in non-metastatic cells 1, protein | 1.499 |
| Ppie | peptidylprolyl isomerase E (cyclophilin E) | 1.501 |
| Ada | adenosine deaminase | 1.502 |
| Fcer2a | Fc receptor, IgE, low affinity II, alpha polypeptide | 1.505 |
| Pim3 | proviral integration site 3 | 1.508 |
| Nup85 | nucleoporin 85 | 1.515 |
| Mt2 | metallothionein 2 | 1.521 |
| Atm | ataxia telangiectasia mutated homolog (human) | 1.522 |
| Hmga2 | high mobility group AT-hook 2 | 1.535 |
| Eif2c2 | eukaryotic translation initiation factor 2C, 2 | 1.535 |
| Tap2 | transporter 2, ATP-binding cassette, sub-family B (MDR/TAP) | 1.536 |
| Tnfaip2 | tumor necrosis factor, alpha-induced protein 2 | 1.536 |
| Bcl2l1 | Bcl2-like 1 | 1.536 |
| Irak1 | interleukin-1 receptor-associated kinase 1 | 1.541 |
| Bak1 | BCL2-antagonist/killer 1 | 1.562 |
| Mark2 | MAP/microtubule affinity-regulating kinase 2 | 1.564 |
| Gss | glutathione synthetase | 1.571 |
| Socs4 | suppressor of cytokine signaling 4 | 1.573 |
| Cxcl2 | chemokine (C-X-C motif) ligand 2 | 1.576 |
| Tlr4 | toll-like receptor 4 | 1.597 |
| Stat6 | signal transducer and activator of transcription 6 | 1.609 |
| Odc1 | ornithine decarboxylase, structural 1 | 1.613 |
| Sfn | stratifin | 1.613 |
| Slc16a3 | solute carrier family 16 (monocarboxylic acid transporters), member 3 | 1.615 |
| Csf2rb2 | colony stimulating factor 2 receptor, beta 2, low-affinity (granulocyte-macrophage) | 1.625 |
| Ddx39 | DEAD (Asp-Glu-Ala-Asp) box polypeptide 39 | 1.635 |
| Mapk14 | mitogen activated protein kinase 14 | 1.651 |
| Il1rl1 | interleukin 1 receptor-like 1 | 1.664 |
| Il15ra | interleukin 15 receptor, alpha chain | 1.666 |
| Ifrd1 | interferon-related developmental regulator 1 | 1.717 |
| Ncam1 | neural cell adhesion molecule 1 | 1.758 |
| Slc6a12 | solute carrier family 6 (neurotransmitter transporter, betaine/GABA), member 12 | 1.775 |
| Pim1 | proviral integration site 1 | 1.783 |
| Adora3 | adenosine A3 receptor | 1.785 |
| Gas7 | growth arrest specific 7 | 1.786 |
| Hspa1b | heat shock protein 1B | 1.787 |
| Mef2d | myocyte enhancer factor 2D | 1.789 |
| Slc25a18 | solute carrier family 25 (mitochondrial carrier), member 18 | 1.791 |
| Ifrd2 | interferon-related developmental regulator 2 | 1.796 |
| Il1rn | interleukin 1 receptor antagonist | 1.801 |
| Vegfa | vascular endothelial growth factor A | 1.82 |
| Tnfrsf9 | tumor necrosis factor receptor superfamily, member 9 | 1.833 |
| S100a10 | S100 calcium binding protein A10 (calpactin) | 1.868 |
| Klra2 | killer cell lectin-like receptor, subfamily A, member 2 | 1.904 |
| Cd14 | CD14 antigen | 1.919 |
| Hif3a | hypoxia inducible factor 3, alpha subunit | 1.924 |
| Pak4 | p21 (CDKN1A)-activated kinase 4 | 1.991 |
| Il18r1 | interleukin 18 receptor 1 | 1.995 |
| Ampd2 | adenosine monophosphate deaminase 2 (isoform L) | 2.058 |
| C2ta | class II transactivator | 2.058 |
| Serpini1 | serine (or cysteine) peptidase inhibitor, clade I, member 1 | 2.066 |
| Il18rap | interleukin 18 receptor accessory protein | 2.134 |
| Il15ra | interleukin 15 receptor, alpha chain | 2.181 |
| Cyp7a1 | cytochrome P450, family 7, subfamily a, polypeptide 1 | 2.193 |
| Hmmr | hyaluronan mediated motility receptor (RHAMM) | 2.225 |
| Irf2 | interferon regulatory factor 2 | 2.244 |
| Cxcl12 | chemokine (C-X-C motif) ligand 12 | 2.283 |
| Ier3 | immediate early response 3 | 2.397 |
| Nr4a1 | nuclear receptor subfamily 4, group A, member 1 | 2.52 |
| Irf7 | interferon regulatory factor 7 | 2.593 |
| Slc17a6 | solute carrier family 17 (sodium-dependent inorganic phosphate cotransporter), member 6 | 6.313 |

**Supplemental Table S1. Microarray analysis of inflammatory gene expression in RAW 264.7 macrophages during incubation in CT26 tumor cell conditioned buffer.** To examine inflammatory gene expression, conditioned buffer was collected from CT26 cultures following 48 hrs of incubation at 37°C and applied to RAW 264.7 cultures for an additional 24 hrs at 37°C. mRNA was then isolated, reverse transcribed and analyzed by hybridization to the Codelink Mammalian Inflammation Bioarray (GE Healthcare, Piscataway, NJ), according to the manufacturer recommendations. Significance of transcript upregulation or downregulation was determined using the VAMPIRE statistical algorithm. Data represents the fold change in transcript expression from 3 separate experiments. Of the 854 genes assayed, 270 genes were determined to be significantly upregulated or downregulated in RAW 264.7 macrophages during culture in CT26 conditioned buffer.
