# Supplementary material for: Chemoattractant Signaling between Tumor Cells and Macrophages Regulates Cancer Cell Migration, Metastasis and Neovascularization
Source: PLoS One. 2009 Aug 21;4(8):e6713. doi: 10.1371/journal.pone.0006713 (PMC2725301; doi:10.1371/journal.pone.0006713)
Supplement: Table S2 — Microarray analysis of inflammatory gene expression in CT26 tumor cells during incubation in RAW 264.7 macrophage conditioned buffer. To examine inflammatory gene expression, conditioned buffer was collected from RAW 264.7 cultures following 48 hrs of incubation at 37°C and applied to CT26 cultures for an additional 24 hrs at 37°C. mRNA was then isolated, reverse transcribed and analyzed by hybridization to the Codelink Mammalian Inflammation Bioarray (GE Healthcare, Piscataway, NJ), according to the manufacturer recommendations. Significance of transcript upregulation or downregulation was determined using the VAMPIRE statistical algorithm. Data represents the fold change in transcript expression from 3 separate experiments. Of the 854 genes assayed, 85 genes were determined to be significantly upregulated or downregulated in CT26 cells during culture in RAW 264.7 conditioned buffer. (0.12 MB DOC) [file pone.0006713.s004.doc]

**Green etal.**

**Supplemental Table S2**

| **Gene** | **Gene Description** | **CB/Ctrl** |
| --- | --- | --- |
| Hck | hemopoietic cell kinase | 0.005 |
| Mark2 | MAP/microtubule affinity-regulating kinase 2 | 0.012 |
| Hspa1b | heat shock protein 1B | 0.274 |
| Fgf10 | fibroblast growth factor 10 | 0.331 |
| Taf1c | TATA box binding protein (Tbp)-associated factor, RNA polymerase I, C | 0.491 |
| Ctgf | connective tissue growth factor | 0.55 |
| Grem1 | gremlin 1 | 0.556 |
| Cd80 | CD80 antigen | 0.564 |
| Hspa1a | heat shock protein 1A | 0.582 |
| Cyp26b1 | cytochrome P450, family 26, subfamily b, polypeptide 1 | 0.607 |
| Hmga2 | high mobility group AT-hook 2 | 0.653 |
| Socs1 | suppressor of cytokine signaling 1 | 0.664 |
| Slc20a1 | solute carrier family 20, member 1 | 0.683 |
| Ifrd2 | interferon-related developmental regulator 2 | 0.689 |
| Eef1e1 | eukaryotic translation elongation factor 1 epsilon 1 | 0.692 |
| Cd14 | CD14 antigen | 0.694 |
| Bcor | Bcl6 interacting corepressor | 1.416 |
| Fosl1 | fos-like antigen 1 | 1.445 |
| Vegfa | vascular endothelial growth factor A | 1.464 |
| Sdc4 | syndecan 4 | 1.465 |
| Slc11a2 | solute carrier family 11 (proton-coupled divalent metal ion transporters), member 2 | 1.476 |
| Tnfrsf9 | tumor necrosis factor receptor superfamily, member 9 | 1.483 |
| Itpk1 | inositol 1,3,4-triphosphate 5/6 kinase | 1.493 |
| Rela | v-rel reticuloendotheliosis viral oncogene homolog A (avian) | 1.499 |
| Fgfr1 | fibroblast growth factor receptor 1 | 1.512 |
| Eef2 | eukaryotic translation elongation factor 2 | 1.524 |
| Tgif | TG interacting factor | 1.525 |
| Traf4 | Tnf receptor associated factor 4 | 1.533 |
| Tgfb3 | transforming growth factor, beta 3 | 1.555 |
| Nfatc1 | nuclear factor of activated T-cells, cytoplasmic, calcineurin-dependent 1 | 1.559 |
| Slc1a5 | solute carrier family 1 (neutral amino acid transporter), member 5 | 1.566 |
| Cxcl10 | chemokine (C-X-C motif) ligand 10 | 1.571 |
| Tbpl1 | TATA box binding protein-like 1 | 1.581 |
| Cebpb | CCAAT/enhancer binding protein (C/EBP), beta | 1.602 |
| Jun | Jun oncogene | 1.62 |
| 6620401K05Rik | RIKEN cDNA 6620401K05 gene | 1.623 |
| Slc7a5 | solute carrier family 7 (cationic amino acid transporter, y+ system), member 5 | 1.735 |
| Smad7 | MAD homolog 7 (Drosophila) | 1.753 |
| Ier5 | immediate early response 5 | 1.753 |
| Nfatc1 | nuclear factor of activated T-cells, cytoplasmic, calcineurin-dependent 1 | 1.757 |
| Tgfb1 | transforming growth factor, beta 1 | 1.777 |
| Arhgef2 | rho/rac guanine nucleotide exchange factor (GEF) 2 | 1.784 |
| Tap1 | transporter 1, ATP-binding cassette, sub-family B (MDR/TAP) | 1.8 |
| Ifngr2 | interferon gamma receptor 2 | 1.821 |
| Ifrd1 | interferon-related developmental regulator 1 | 1.822 |
| Socs2 | suppressor of cytokine signaling 2 | 1.852 |
| Daxx | Fas death domain-associated protein | 1.878 |
| Ralgds | ral guanine nucleotide dissociation stimulator | 1.926 |
| Nfkbib | nuclear factor of kappa light chain gene enhancer in B-cells inhibitor, beta | 1.933 |
| Irf1 | interferon regulatory factor 1 | 1.937 |
| Map3k14 | mitogen-activated protein kinase kinase kinase 14 | 1.996 |
| Klf7 | Kruppel-like factor 7 (ubiquitous) | 2.002 |
| Arrb2 | arrestin, beta 2 | 2.004 |
| Pim3 | proviral integration site 3 | 2.032 |
| Gadd45b | growth arrest and DNA-damage-inducible 45 beta | 2.055 |
| Bcl10 | B-cell leukemia/lymphoma 10 | 2.121 |
| Nfkb2 | nuclear factor of kappa light polypeptide gene enhancer in B-cells 2, p49/p100 | 2.145 |
| Zfp36 | zinc finger protein 36 | 2.147 |
| Il15ra | interleukin 15 receptor, alpha chain | 2.201 |
| Birc3 | baculoviral IAP repeat-containing 3 | 2.211 |
| Nfe2l1 | nuclear factor, erythroid derived 2,-like 1 | 2.265 |
| Relb | avian reticuloendotheliosis viral (v-rel) oncogene related B | 2.408 |
| Tnip1 | TNFAIP3 interacting protein 1 | 2.584 |
| Csf1 | colony stimulating factor 1 (macrophage) | 2.63 |
| Nfkbia | nuclear factor of kappa light chain gene enhancer in B-cells inhibitor, alpha | 2.696 |
| Traf1 | Tnf receptor-associated factor 1 | 2.834 |
| Vcam1 | vascular cell adhesion molecule 1 | 2.971 |
| Gadd45a | growth arrest and DNA-damage-inducible 45 alpha | 3.067 |
| Cxcl1 | chemokine (C-X-C motif) ligand 1 | 3.149 |
| Ccl2 | chemokine (C-C motif) ligand 2 | 3.173 |
| Mmp10 | matrix metallopeptidase 10 | 3.261 |
| Egr2 | early growth response 2 | 3.406 |
| Junb | Jun-B oncogene | 3.571 |
| Ccl20 | chemokine (C-C motif) ligand 20 | 3.802 |
| Alk | anaplastic lymphoma kinase | 3.822 |
| Nfkbie | nuclear factor of kappa light polypeptide gene enhancer in B-cells inhibitor, epsilon | 4.786 |
| Egr1 | early growth response 1 | 5.073 |
| Csf2 | colony stimulating factor 2 (granulocyte-macrophage) | 5.101 |
| Saa3 | serum amyloid A 3 | 5.338 |
| Cxcl2 | chemokine (C-X-C motif) ligand 2 | 6.663 |
| Mtap7 | microtubule-associated protein 7 | 7.513 |
| Sh2d1a | SH2 domain protein 1A | 7.65 |
| Ifna9 | interferon alpha 9 | 9.261 |
| Cyp2s1 | cytochrome P450, family 2, subfamily s, polypeptide 1 | 9.864 |
| Fcer1g | Fc receptor, IgE, high affinity I, gamma polypeptide | 12.513 |
| Ccl11 | small chemokine (C-C motif) ligand 11 | 570.566 |

**Supplemental Table S2. Microarray analysis of inflammatory gene expression in CT26 tumor cells during incubation in RAW 264.7 macrophage conditioned buffer.** To examine inflammatory gene expression, conditioned buffer was collected from RAW 264.7 cultures following 48 hrs of incubation at 37°C and applied to CT26 cultures for an additional 24 hrs at 37°C. mRNA was then isolated, reverse transcribed and analyzed by hybridization to the Codelink Mammalian Inflammation Bioarray (GE Healthcare, Piscataway, NJ), according to the manufacturer recommendations. Significance of transcript upregulation or downregulation was determined using the VAMPIRE statistical algorithm. Data represents the fold change in transcript expression from 3 separate experiments. Of the 854 genes assayed, 85 genes were determined to be significantly upregulated or downregulated in CT26 cells during culture in RAW 264.7 conditioned buffer.
